# Divergent FOXA1 mutations drive prostate tumorigenesis and therapy-resistant cellular plasticity

**DOI:** 10.1126/science.adv2367

**Published:** 2025-09-04

**Authors:** Sanjana Eyunni, Rahul Mannan, Yuping Zhang, Eleanor Young, Qiuyang Zhang, Jie Luo, Matthew Pang, Somnath Mahapatra, Jean Ching-Yi Tien, James M. George, Mustapha Jaber, Hamzah Hakkani, Sandra E. Carson, Abigail J. Todd, Noshad Hosseini, Mahnoor Gondal, Ryan J. Rebernick, Xuhong Cao, Fengyun Su, Rui Wang, Rohit Mehra, Jing Li, Marcin Cieslik, Arul M. Chinnaiyan, Abhijit Parolia

**Affiliations:** 1 Department of Pathology, University of Michigan; Ann Arbor, MI, USA.; 2 Michigan Center for Translational Pathology, University of Michigan; Ann Arbor, MI, USA.; 3 Molecular and Cellular Pathology Program, University of Michigan; Ann Arbor, MI, USA.; 4 Department of Computational Medicine and Bioinformatics, University of Michigan; Ann Arbor, MI, USA.; 5 Howard Hughes Medical Institute, University of Michigan; Ann Arbor, MI, USA.; 6 Rogel Cancer Center, University of Michigan, Ann Arbor; MI, USA.; 7 Department of Precision Medicine, Changhai Hospital, Second Military Medical University (Naval Medical University); Shanghai, China.; 8 Department of Urology, University of Michigan; Ann Arbor MI, USA.

## Abstract

FOXA1 is altered in 10–40% of prostate cancers, yet its oncogenic mechanisms remain uncharacterized *in vivo*. We developed knock-in mouse models representing distinct classes of FOXA1 mutations. Histopathological and multi-omic analyses of prostate tissues and organoids revealed that Class 1 mutations, in conjunction with *p53* inactivation, drive androgen-dependent adenocarcinomas through co-activation of mTORC1/2 and oncogenic AR signaling stemming from chimeric AR-half enhancers. In contrast, Class 2 mutations induce intra-luminal plasticity by reprogramming differentiated luminal cells into a progenitor-like state through activation of KLF5 and AP-1 neo-enhancer circuitries, which enables enhanced survival and proliferation even under castrate androgen levels. Our findings establish FOXA1 as a multifaceted oncogene, with distinct mutational classes divergently evolving to drive prostate tumorigenesis or therapy-resistant progression.

## One line summary

Distinct FOXA1 mutations reprogram prostate luminal epithelial cell fate, driving either carcinogenesis or therapy resistance.

Prostate cancer (PCa) is characterized by transcriptional dysregulation, with over 70% of the primary disease driven by somatic alterations affecting transcription factors (TFs) and their regulatory chromatin and epigenetic cofactors (1–3). Activating ETS family gene fusions with highly active promoters are pathognomonic driver alterations in primary PCa—recurrent in 50–60% of cases in European White men (4). Genomic studies have also uncovered frequent alterations in the pioneer TF FOXA1 in 10–35% of PCa in this population (2, 3, 5–7). In the Asian population, however, FOXA1 mutations are detected in over 40% of primary prostatic tumors, surpassing ETS fusions to become the predominant driver alteration in this patient ethnicity (8).

Almost all primary PCa express the lineage-specific androgen receptor (AR) protein. AR activity undergoes extensive reprogramming during prostate tumorigenesis, resulting in acquisition of oncogenic functions (9, 10)essential for hyperproliferation and survival of tumor cells (11). Thus, targeted therapies blocking either the production of androgen (i.e., surgical or medical castration) or directly inhibiting AR (e.g. enzalutamide, etc.) comprise mainstay regimens after surgical resection or radiation of the localized disease (12, 13). Androgen deprivation therapies (ADTs) induce only temporary disease remission and, in all treated patients, the tumor relapses by further acquiring mutations in AR pathway genes that restore its activity. The recurrent disease is clinically referred to as castration-resistant prostate cancer (CRPC) (14, 15).

**Fig. 1.**
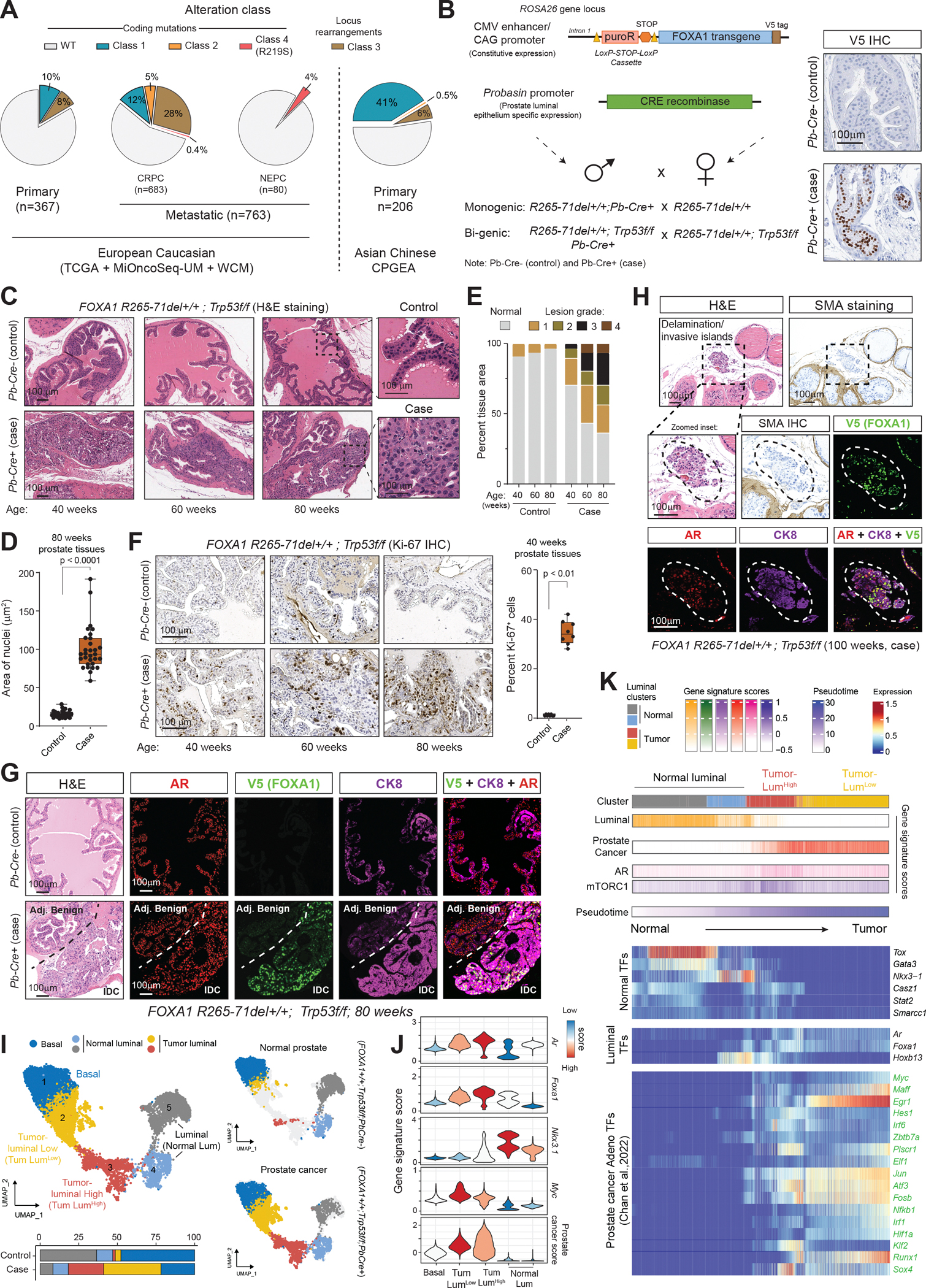
FOXA1 Class 1 mutants drive high-grade invasive adenocarcinoma in the background of p53 loss. **A)** Frequency of FOXA1 alterations in the European (primary and metastatic) and Asian (primary) prostate cancer patient cohorts. FOXA1 Class 1 mutations are truncal alterations seen in the primary and metastatic disease (10–12%) and comprise in-frame indels and missense mutations in the wing2 region of the protein. Class 2 mutations are acquired in the metastatic disease and comprise truncating frameshift alterations in the C-terminal region of the protein. Class 3 mutations are structural alterations (duplications and translocations) within the *FOXA1* syntenic loci found in the primary and metastatic disease (8–28%). FOXA1 Class 4 mutations comprise the missense R219S alteration that is primary enriched in NEPC tumors (4%). Indel, insertion, and deletion; CRPC, castration-resistant prostate cancer; NEPC, neuroendocrine prostate cancer; U-M, University of Michigan; WCM, Weill Cornell Medicine; CPGEA: Chinese Prostate Cancer Genome and Epigenome Atlas. B) Left: Schematic of FOXA1 Class 1 (*R265–71del*) knock-in transgenic mouse models and mating strategies for monogenic (*R265–71del+/+*) and bigenic (*R265–71del+/+; Trp53f/f*) mouse lines. Probasin-Cre (Pb-Cre) ensures prostate-specific expression of FOXA1. Control and case animals are denoted as *Pb-Cre−* and *Pb-Cre+,* respectively. Right: V5-epitope tag immunohistochemistry (IHC) in control (*Pb-Cre−*) and case (*Pb-Cre+*) *R265–71del+/+* animals. Scale=100μm. C) Representative Hematoxylin and Eosin (H&E) stained cross-sections of *FOXA1 R265–71del+/+;Trp53f/f* control and case prostate tissues (anterior lobe) from 40 to 80 week-old mice. Black-dotted boxes denote zoomed insets. Scale=100μm. D) Area of nuclei in 80-week-old control and case *FOXA1 R265–71del+/+;Trp53f/f* prostate tissues (two-tailed t-test). E) Histopathological grading of tissues from panel c. Grade 1: Hyperplasia, Grade 2: Low/high-grade prostate intraepithelial neoplasia (PINs), Grade 3: Florid high-grade PINs and/or Atypical Intraductal Proliferation (AIP) and/or Intraductal carcinoma (IDC), Grade 4: Invasive adenocarcinoma. F) Left: Ki67 IHC in control and case *FOXA1 R265–71del+/+;Trp53f/f* prostate tissues at 40, 60, and 80 weeks. (Scale=100μm). Right: Statistical representation of quantification (percentage of KI67-positive cells) from 40-week-old tissues from the left panel (two-tailed t-test). Box plot: center line, median; box, interquartile range (Q1–Q3); whiskers, minimum to maximum values; all individual data points shown. G) Representative H&E and multiplex immunofluorescence of V5, AR, and CK8 in *FOXA1 R265–71del+/+;Trp53f/f* 80-week-old prostate tissues. Adjacent benign and intraductal carcinoma (IDC) tumor regions are marked. Scale=100μm. H) Representative H&E, alpha-smooth muscle actin (SMA) IHC and multiplex immunofluorescence of noted proteins in *FOXA1 R265–71del+/+;Trp53f/f* 100-week-old tissues. Invasive islands denoting breach of basement membrane (lack of SMA) are highlighted in a black, dotted box. Scale=100μm. I) Left: Uniform Manifold Approximation and Projection (UMAP) plots from single-cell RNA-seq data of *FOXA1 R265–71del+/+;Trp53f/f* 100-week-old anterior and dorsal prostate lobes. Tumor cell clusters are highlighted in yellow and red. Right: Split UMAP plots with only case or control tissue-derived cells colored across clusters. J) Violin plots showing expression of *Ar, Foxa1, Nkx3.1, Myc* and a prostate cancer score (defined in this paper; see Methods) in distinct cell populations described in panel I. K) Heatmap of select transcription factor (TF) gene expression along the pseudotime (Slingshot, see Methods) from normal luminal cells to tumor cells derived from *FOXA1 R265–71del+/+;Trp53f/f* control and case prostate tissues. The top heatmaps show aggregate z-scores of luminal, AR, mTORC1, and prostate cancer signature genes.

As a pioneer factor, FOXA1 binds to nucleosomal DNA at enhancer sites and triggers chromatin decompaction to enable transcriptional activity of lineage-specific TFs (16, 17), including AR in the prostate luminal epithelia (18, 19). Our group recently characterized FOXA1 alterations into three distinct structural classes, each showing disparate clinical incidence and molecular gain-of-functions (5) (Fig. 1A). Class 1 alterations are detected in localized, primary tumors that primarily comprise missense and in-frame indel mutations in the Wing 2 region of the DNA-binding forkhead domain, imparting FOXA1 higher nuclear mobility and transactivation potential for oncogenic AR signaling (5). In contrast, FOXA1 Class 2 mutations are clonally detected only in the metastatic disease and comprise frameshift mutations in the C-terminal half of the protein. Class 2 mutants show dominance in engaging chromatin and de-repressing metastatic WNT signaling (5). Class 3 alterations comprise structural variations within the *FOXA1* gene locus that amplify a conserved enhancer to increase wild-type FOXA1 activity. Similar FOXA1 alterations are also detected in other hormone-driven malignancies, including breast, salivary gland, and bladder tumors (20–24).

Despite such high clinical recurrence of FOXA1 alterations, their pathobiology remains unexplored in mouse models. In this study, we present genetically engineered mice with targeted knock-in of human cancer-associated FOXA1 mutant transgenes, which provide crucial mechanistic insights into the divergent mechanisms of FOXA1 Class 1 and Class 2 alterations in PCa formation and progression.

## FOXA1 Class 1 mutation with *Trp53* deletion drives high-grade invasive adenocarcinoma

We first engineered a transgenic mouse carrying the human *FOXA1 R265–71del* (Class 1) mutant transgene in the *Rosa26* locus. The knock-in sequence consisted of a constitutive promoter driving expression of the LoxP-flanked puromycin-resistance (puroR) selection marker, followed by the FOXA1-mutant coding sequence (Fig. 1B, fig. S1A). We crossed FOXA1 transgenic female mice with Pb-Cre4 males (25) to obtain *FOXA1;Pb-Cre* offspring. *FOXA1+/+;Pb-Cre+* males were denoted as “case” while littermate *FOXA1+/+;Pb-Cre−* males served as controls (Fig. 1B). As expected, we saw specific and robust nuclear staining of V5 in prostate epithelial cells in case relative to the control prostate tissue (Fig. 1B), which looked histologically normal at 10–12 weeks of age (fig. S1B). However, histopathological evaluation of older animals revealed Class 1 mutants to drive hyperplasia in the epithelial lining, with intraepithelial neoplasia spanning 20–25% of the tissue by 60 weeks of age. We also detected focal high-grade, Ki67-positive intra-ductal carcinomas (IDCs) in 25% of the cases (fig. S1C–E), with similar lesions being undetectable in control tissues.

Next, assessment of genomic co-alterations from metastatic tumors uncovered Class 1 mutations to frequently co-occur with biallelic loss of *TP53* while being mutually exclusive to *PTEN* deletions (fig. S1F). Thus, we generated compound two-gene models by crossing FOXA1 Class 1 mice with mice harboring floxed, inactivating alleles of the *Trp53* tumor suppressor gene (Fig. 1B, fig. S1G). In prostate tissues from the *FOXA1 R265–71del+/+;Trp53f/f;Pb-Cre+* case animals, we detected widespread hyper-stratified epithelial lesions with enlarged nuclei in the anterior and dorsal glands starting around the 40-week timepoint (Fig. 1C,D). With age, cancerous lesions expanded to cover a larger tissue area and exhibited characteristics of high-grade disease (Fig. 1C,E), assessed using our in-house grading system (see Methods and fig. S1H). Ki67 staining revealed 30–40% of the cells within these lesions to be actively proliferating (Fig. 1F, fig. S1I). At 80 weeks, case animals had enlarged urogenital tracts (fig. S1J), and the prostate tissues showed expansive, luminous glandular structures that were encapsulated by high-grade intraductal adenocarcinoma (fig. S1K). These lesions expressed the V5-tagged FOXA1 R265–71del mutant along with AR and CK8 luminal markers (Fig. 1G), akin to primary prostatic tumors detected in humans. Starting around 60 weeks of age, *R265–71del+/+;Trp53f/f;Pb-Cre+* mice also showed signs of invasion of the adenocarcinoma into the surrounding stromal regions (denoted as grade 4 lesions; Fig. 1E and fig. S2A). These invasive lesions were identified by breach of the surrounding SMA-positive fibromuscular layer and detection of delaminated stromal islands of tumor cells expressing CK8, AR, and transgenic FOXA1 (Fig. 1H, fig. S2B). In a single 100-week-old case animal, we also detected a large tumor mass with adenocarcinoma histology and robust expression of transgenic FOXA1 mutant protein (fig. S2C,D).

Next, to mechanistically characterize the *R265–71del+/+;Trp53f/f* prostatic tumors, we performed single-cell RNA-sequencing of anterior and dorsal prostate lobes harvested from 100-week-old case and control animals (fig. S3A). We isolated clusters containing epithelial cells where, as expected, we found a specific loss of the puroR transcript in Cre-expressing cells derived from case animals (fig. S3B,C). Consistent with histological data, *R265–71del+/+;Trp53f/f;Pb-Cre+* prostate tissues had 2-fold higher epithelial cell fraction in S phase (DNA replication) of the cell cycle, indicating hyperproliferation (fig. S3D). Louvain clustering identified five distinct epithelial cell clusters, with clusters 2 and 3 comprising cells predominantly coming from the cancerous tissues (Fig. 1I). These cell clusters showed highest expression of both *Ar* and *Foxa1* genes (Fig. 1J). Simultaneously, these tumor cells showed a significant downregulation of the tumor suppressor gene *Nkx3.1* and aberrant upregulation of *Myc* (Fig. 1J). We were also able to detect a decrease in NKX3.1 protein levels within PCa lesions relative to the adjacent normal epithelia (fig. S3E). Based on expression levels of Ar and Foxa1, we labeled the two tumor-specific epithelial clusters as luminal high (cluster 3) and low (cluster 2; Fig. 1I,J). Through differential expression analyses, we also defined a 50-gene signature of FOXA1 Class 1-driven mouse prostate adenocarcinoma that was significantly activated in tumor cells (fig. S3F). Cross-species analyses showed a significant enrichment of this mouse PCa gene signature in human Class 1-mutant PCa specimens, and reciprocally human Class 1-mutant gene signature was enriched in mouse prostatic tumors (fig. S3G,H). Pathway analyses demonstrated conserved activation of hyperproliferative programs—including E2F/G2M cell cycle and MYC signaling—as well as activation of luminal androgen response and PI3K-mTORC1 pathways in both human and mouse Class 1-driven tumors (fig. S3I).

Next, to characterize sequential changes in the transcriptional landscape during transformation, we performed Slingshot pseudotime trajectory analysis (fig. S3J). This confirmed loss of *Nkx3.1* expression upon emergence of PCa with a parallel increase in *Ar* and *Foxa1* levels (Fig. 1K). We also detected an aberrant gain of a host of AR+ adenocarcinoma-associated TFs that were recently identified in other murine PCa models (26) (Fig. 1K). Altogether, through detailed histopathological and single-cell transcriptomic characterization, we show that overexpression of FOXA1 Class 1 mutants along with p53 inactivation in the prostate epithelia triggers formation of AR+ adenocarcinoma that strongly recapitulates luminal features of primary human disease. This also constitutes primary experimental evidence causally linking FOXA1 Class 1 mutations to prostate tumorigenesis, thereby substantiating these early genetic alterations as bona fide drivers of human PCa.

## FOXA1 Class 1 mutants co-activate AR and mTORC1/2 tumorigenic pathways

**Fig. 2.**
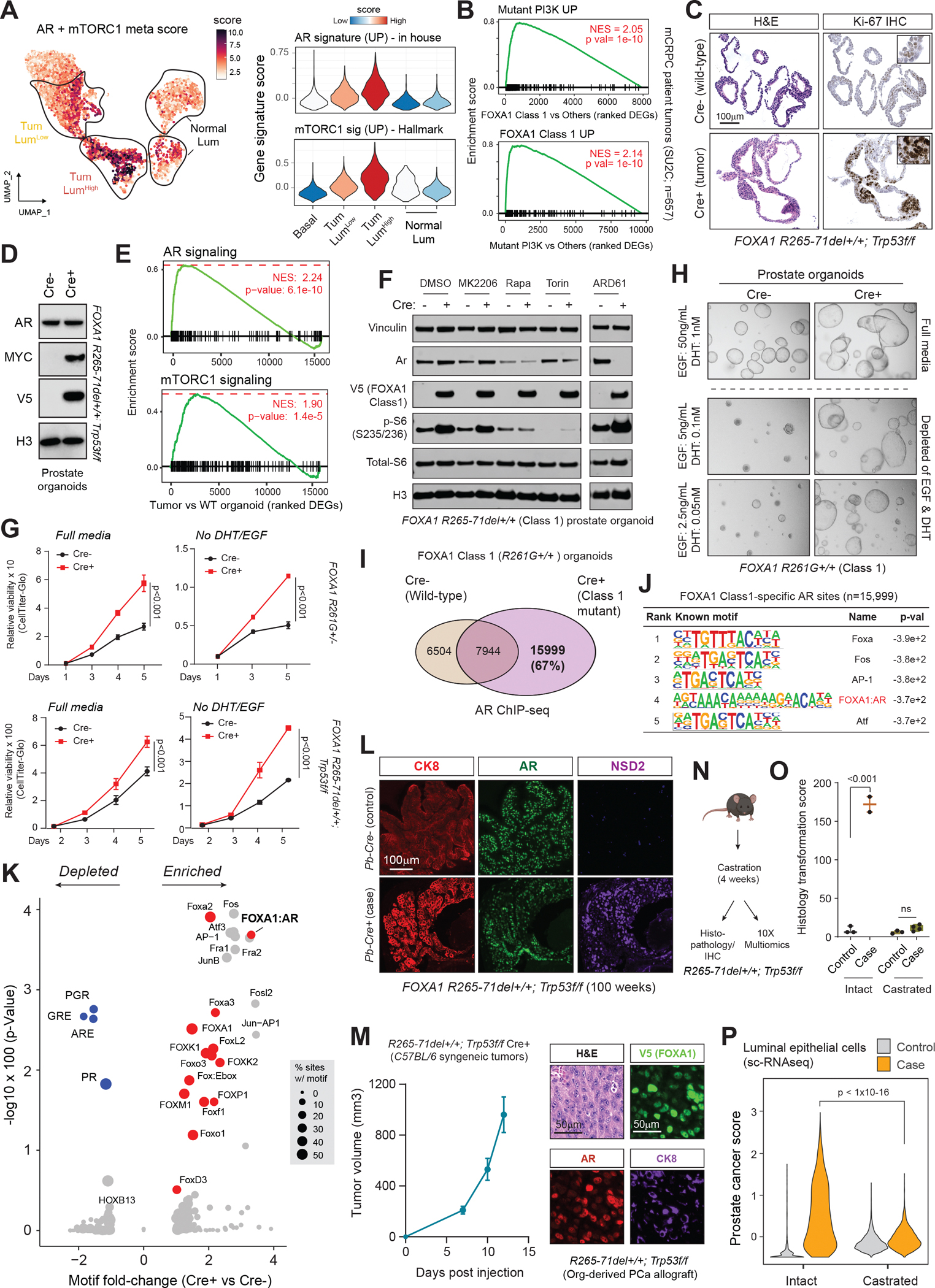
FOXA1 Class 1 mutants co-activate AR and mTORC1 oncogenic signaling. **A)** Left: UMAP plots from single-cell RNA-seq data of *R265–71del+/+;Trp53f/f* tissues showing the meta-score of AR and mTORC1 gene signatures. Right: Violin plots of AR and mTORC1 gene signature scores in the tumor and normal clusters. B) Gene set enrichment analysis (GSEA) plots for PI3K and FOXA1 Class 1 activated genes from FOXA1 Class 1 or PI3K mutant patient tumor tissues. DEGS, differentially expressed genes (GSEA enrichment test). C) Representative H&E and Ki67 IHC in Class 1 *R265–71del+/+;Trp53f/f* isogenic organoids. *Cre−* organoids are wild-type/normal organoids, while *Cre+* organoids are tumor organoids. Scale=100μm. D) Immunoblot of labeled proteins in the Cre− and Cre+ tumor organoids. Total H3 is used as a loading control. E) GSEA plots of AR and mTORC1 up-regulated genes using the fold change from *FOXA1 R265–71del+/+;Trp53f/f* Cre+ vs Cre− tumor organoids. DEGS, differentially expressed genes. (n=2 biological replicates, GSEA enrichment test). F) Immunoblot of labeled proteins in *R265–71del+/+* Cre− and Cre+ organoids treated with MK2206 (AKT inhibitor), rapamycin (mTORC1 inhibitor), torin (mTORC1/2 inhibitor), or ARD61 (AR degrader) for 24 hours. G) Growth curves (cell titer glow) of Class 1 single gene (*R261G+/*−) and two-gene (*R265–71del+/+; Trp53f/f*) Cre− and Cre+ organoid lines grown in full media or media lacking DHT and EGF (n=4 biological replicates, two-sided t-test, mean with SD is shown). H) Representative images of Class 1 (*R261G+/+*) Cre− and Cre+ organoids grown in complete media or media depleted of EGF and DHT. I) Venn diagram showing overlaps of AR ChIP-seq in Cre− and Cre+ Class 1 *R261G+/+* organoids. J) Top five known HOMER motifs (ranked by p-value) enriched within Cre+, i.e., Class 1 specific AR binding sites. (HOMER, hypergeometric test). K) Fold change and significance of HOMER motifs enriched within Cre+ AR sites over Cre− AR binding sites (HOMER, hypergeometric test). L) Representative multiplex-IF images of CK8, AR, and NSD2 in anterior lobes of FOXA1 Class 1 *R265–71del+/+;Trp53f/f* tumors. Scale = 100μm. M) Left: Tumor volumes of syngeneic *R265–71del+/+;Trp53f/f* Cre+ tumor organoids grafted in the subcutaneous flanks of *C57BL/6* mice. Right: Representative H&E and multiplex-IF of labeled proteins in tumor allografts obtained from the left panel. Scale =50μm. N) Schematic of castration experiment performed in 60-week-old *FOXA1 R265–71del+/+;Trp53f/f* mice. O) Transformation score (defined in this paper, see methods) in 60-week-old intact and castrated *FOXA1 R265–71del+/+;Trp53f/f* prostate tissues (two-sided t-test). Box plot: center line, median; box, interquartile range (Q1–Q3); whiskers, minimum to maximum values; all individual data points shown. P) Prostate cancer score in single-cell RNA-seq from 60-week-old intact and castrated *FOXA1 R265–71del+/+;Trp53f/f* prostate tissues (two-sided Wilcoxon test).

For mechanistic interrogations, we first derived pure, pseudo-bulk transcriptional profiles of normal and cancerous epithelial cells from case and control prostate tissues (see Methods). As expected, gene set enrichment analysis of genes differentially expressed between cancer and normal luminal epithelia showed significant activation of hyper-proliferative and other cancer-associated pathways (fig. S4A), while showing no change in basal cells (fig. S4A). Notably, we found a significant and parallel up-regulation of both AR and mTORC1/2 signaling in tumor cells, as evidenced by the higher AR+mTORC1 meta-scores (Fig. 2A). To further evaluate this in human clinical specimens, we compared bulk transcriptomes in Caucasian primary (TCGA, n=503) and CRPC tumors (SU2C cohort, n=657) (3) harboring Class 1 mutations or PI3K pathway alterations (latter mostly comprising PTEN deletions). Our analysis revealed a significant transcriptomic concordance between the two genomic subtypes (fig. S4B–D), showing reciprocal enrichment of associated gene signatures (Fig. 2B). Similarly, integrative analyses of Asian primary PCa (CPGEA cohort; n=206), where FOXA1 Class 1 is a predominant driver alteration in over 41% of the cases (Fig. 1A, table S1), revealed a strong activation of AR signaling in Class 1-mutant tumors (fig. S4E). We also saw a significant concordance in differentially expressed genes between the Class 1-mutant and PI3K-altered tumor subtypes (fig. S4F,G). These findings suggest that concomitant activation of AR and PI3K-mTORC1/2 growth signaling is a salient feature of FOXA1 Class 1-driven carcinogenesis in both human and mouse prostate tissues.

For further experimental interrogations, we established isogenic control (Cre−) and Cre recombinase-transduced (Cre+) prostate epithelial organoids from *FOXA1 R265–71del+/+;Trp53f/f* transgenic animal (fig. S4H). Compared to the Cre− wild-type organoids, *R265–71del+/+;Trp53f/f;Cre+* organoids showed hyper-stratification of the epithelial lining and a significantly higher proportion of Ki67-positive cells (Fig. 2C), while retaining their cystic morphology. Transcriptomic profiling revealed activation of several hallmark cancer pathways, which were concordantly activated in mouse PCa lesions (fig. S4I,J). This included increased expression of MYC and its downstream oncogenic gene programs associated with malignant transformation (Fig. 2D, fig. S4I). Even in the organoid models, we found significant co-activation of AR and mTORC1 signaling (Fig. 2E). Next, we generated FOXA1 Class 1 mutant ChIPseq data and, using the GREAT algorithm (27), defined its putative gene targets. (fig. S5A). Upon intersection with genes that were significantly up-regulated in either Class 1-mutant *R261G+/+* prostate organoids (used to generate FOXA1 ChIPseq) or *R265–71del+/+;Trp53f/f;Cre+* tumor line (fig. S5B), we identified 141 likely direct targets of FOXA1 Class 1 mutants (table S2). Enrichr pathway analysis (28) of this gene set showed a significant enrichment of many cancer-associated pathways, including epithelial-mesenchymal transitions (EMT), hypoxia, glycolysis, and mTORC1 signaling (fig. S5C).

To discern specific contributions of Class 1 mutations and p53 inactivation in driving tumorigenesis, we employed the *FOXA1 R261G* and *R265–71del* single-gene organoids. Here, overexpression of Class 1 mutants alone was sufficient to parallelly activate both AR and mTORC1 pathways (fig. S5D,E), along with other oncogenic gene programs (fig. S5F). However, p53 inactivation alone failed to significantly modulate these pathways (fig. S5F). Histological assessment of organoid morphology also showed hyper-stratification of the normal epithelial lining only in the compound *FOXA1 R261G+/*−*;Trp53-KO* models (fig. S5G). p53-null organoids more closely resembled the histology of wild-type (i.e., Cre−) normal models. *R261G+/*−*;Trp53-KO* organoids also had the highest percentage of Ki67-positive cells and showed the fastest growth compared to wild-type controls and FOXA1 R261G-expressing lines (fig. S5H–J). This strongly implicates FOXA1 Class 1 mutations to initiate malignant transformation and subsequent loss of *Trp53* to act as a promoting event in PCa development.

Expression of Class 1 mutants in organoids also led to a strong and dose-dependent increase in pS473-AKT and pS6 (S235/236), which are direct substrates of mTORC1/2 kinases (29, 30) (Fig. 2F, fig. S5K). This was corroborated in human specimens, where primary PCa from both Caucasian (2) and Asian (31) men showed significantly elevated levels of phosphorylated RPS6 at serine 235/236 and 240/244 residues in Class 1-mutant tumors compared to other cases (fig. S5L,M). Treatment with mTORC1/2 inhibitors blocked Class 1-induced p-S6 accumulation, but treatment with an AKT inhibitor (MK2206) had little to no effect (Fig. 2F). AR degradation also did not affect p-S6 induction (Fig. 2F), while wild-type FOXA1 overexpression was sufficient to increase p-S6 levels (fig. S5K). Next, in growth assays, both single-gene and compound Class 1 organoids grew faster than their wild-type counterparts in complete media. This enhanced proliferation of Class 1-mutant organoids was even more profound in media lacking ligands of AR and PI3K/mTORC1 pathways, namely DHT and EGF (Fig. 2G,H). Altogether, these data suggest that FOXA1 Class 1 mutants function as hypermorphs and activate mTORC1/2 signaling independently of AKT as well as AR activities.

Next, to assess how Class 1 mutants modulate AR signaling, we performed AR ChIP-seq in Class 1-mutant or wild-type prostate organoids. In presence of the Class 1 mutant, we detected extensive reprogramming of the AR cistrome, with almost 16,000 new *cis*-regulatory elements gained within distal and intronic regions (Fig. 2I, fig. S6A). HOMER motif analysis of Class 1-pioneered AR sites uncovered an enrichment of FOXA1 and chimeric FOXA1:AR-half motifs (Fig. 2J,K), while the normal AR cistrome contained palindromic androgen response elements (ARE) (Fig. 2K, fig. S6B,C). A recent study from our group identified chimeric AR half-sites as a distinctive feature of tumor-specific AR enhancer circuitries, which are activated by the abnormal gain of NSD2 methyltransferase activity (10). Consistently, we found induction of NSD2 expression in Class 1-driven PCa lesions, with no detectable expression in the normal epithelium (Fig. 2L, fig. S6D). NSD2 levels were further elevated within invasive edges and delaminated stromal islands of tumor cells (fig. S6E). In tumor organoids, proximity ligation assay revealed strong interaction between NSD2 and AR proteins (fig. S6F–I). CRISPR-mediated knock-out (KO) of NSD2 in the tumor organoids attenuated their growth and significantly dampened AR signaling (fig. S6J,K) via disruption of AR chromatin loading at over 85% of its binding sites (fig. S6L,M). HOMER motif analysis of the NSD2-dependent AR sites uncovered FOXA1:AR-half chimeric motif as the topmost significantly enriched DNA sequence, while the NSD2-independent sites harbored canonical AREs (fig. S6N,O). Consistently, we also detected a significant decrease in NSD2-catalyzed H3K36me2 ChIP-seq signal at chimeric AR-half sites in the NSD2-KO lines (fig. S6P). These findings are consistent with our prior observations in human PCa (10), and suggest that FOXA1 Class 1 mutants pioneer neo-enhancer elements of AR that harbor non-palindromic, chimeric AR-half motifs and, in concert with NSD2, activate a distinct luminal gene program of prostate tumorigenesis

Further validating their malignant phenotype, *FOXA1 R265–71del+/+;Trp53f/f;Pb-Cre+* tumor organoids exclusively formed large tumors upon subcutaneous implantation into NSG mice (fig. S7A). Tumor organoids also established syngeneic allografts in C57BL/6 host mice while retaining their adenocarcinoma histology and expression of transgenic FOXA1, AR, and CK8 luminal markers (Fig. 2M). To assess if Class 1-driven tumors were sensitive to ADT, we surgically castrated 50–65-week-old *R265–71del+/+;Trp53f/f* case and control animals (n=3–4 per genotype), followed by histological grading and single-cell RNA sequencing of residual tissues four weeks later (Fig. 2N). In contrast to widespread tumors detected in the hormonally-intact case animals, castrated animals had no atypical lesions that stained for Ki67 (Fig. 2O, fig. S7B,C). More compelling, epithelial cells from case tissues showed a significant loss of the Class 1 PCa gene signature upon castration relative to the intact condition (Fig. 2P). Altogether, our findings show that FOXA1 Class 1 mutations promoted by p53 inactivation trigger the formation of high-grade AR+ prostate adenocarcinoma that retain luminal features and regress upon castration, recapitulating cardinal characteristics of the primary human disease.

## FOXA1 Class 2 mutations trigger castration-associated intra-luminal plasticity

**Fig. 3.**
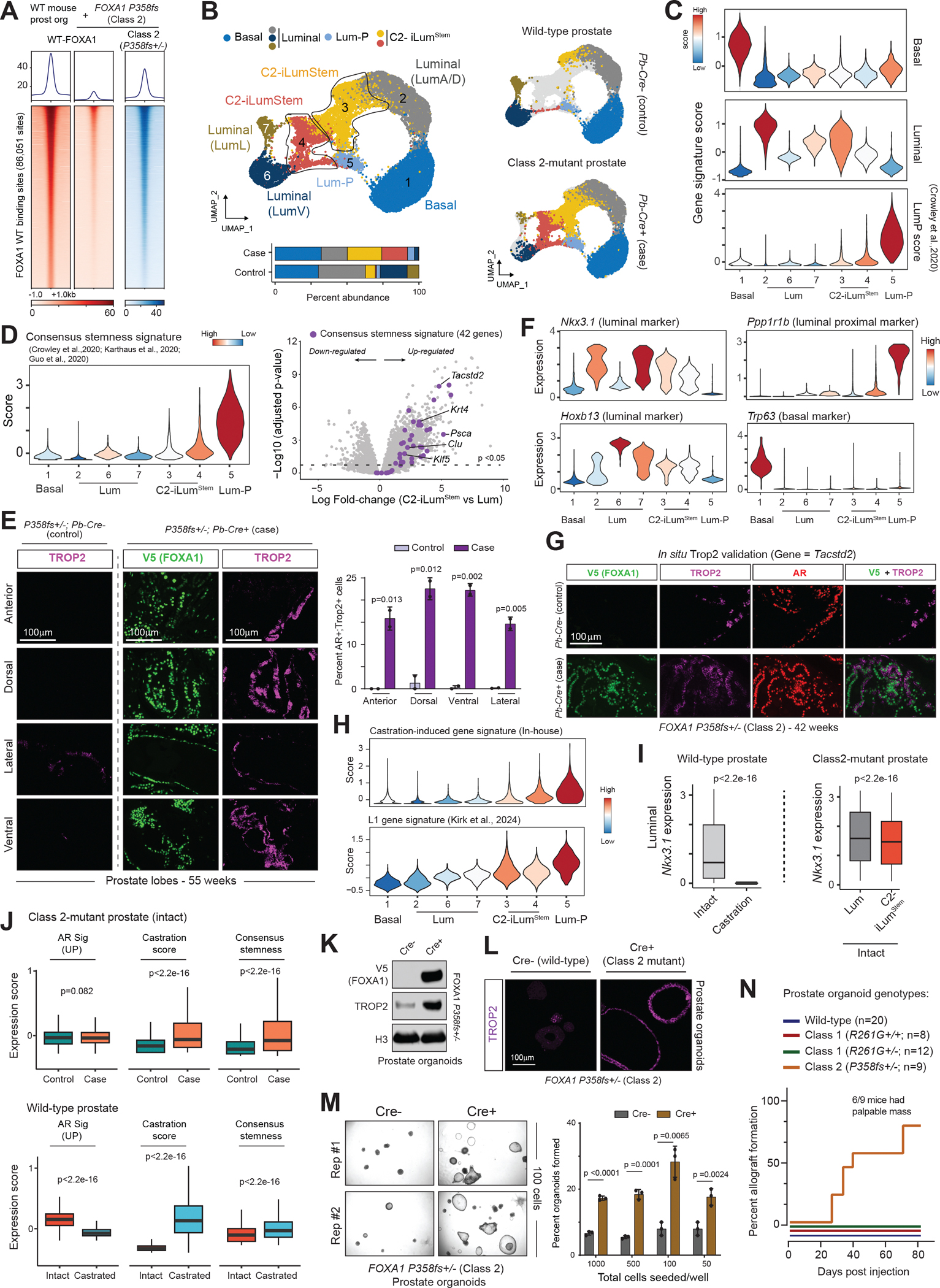
FOXA1 Class 2 mutants trigger castration-associated intra-luminal plasticity. **A)** ChIP-seq read-density heatmaps of FOXA1 wild-type and Class 2 mutant at 86,000 sites in Class 2 *P358fs+/*− Cre− and Cre+ organoids. B) Left: UMAP plots of young and old Class 2 *P358fs+/*− mouse prostate tissues. Two distinct case-specific populations are highlighted in yellow and red. Right: Split UMAP plots with only case or control tissue-derived cells colored across clusters. C) Violin plots of noted signatures in the distinct clusters emanating from the Class 2 case and control prostate tissues. D) Left: UMAP plots of the consensus stemness signature (see Methods) in the Class 2 case and control clusters. Right: Volcano plot of up and down-regulated genes from the case vs control Class 2 single-cell RNA-seq data. Consensus stemness genes are highlighted as purple dots. E) Left: Representative multiplex-IF stained sections of V5 and TROP2 in distinct lobes of 55-week-old Class 2 mutant tissues (*Pb-Cre*− represents control tissues, and *Pb-Cre+* represents case tissues). Scale =100μm. Right: Percentage of AR+/TROP2+ cells in distinct lobes of control and case Class 2 mutant tissues (two-sided t-test). F) Violin plots showing mRNA expression (imputed) of noted genes in the distinct Class 2 case and control-specific clusters. G) Representative multiplex-IF stained sections of V5, TROP2, and AR in 42-week-old Class 2 mutant case and control tissues. Scale=100μm. H) Violin plots of the castration-induced gene signature (defined in-house, see methods) and an L1 gene signature (defined by Kirk et al., 2024) in the Class 2 case and control clusters. I) Boxplots of *Nkx3.1* mRNA expression in intact and castrated wild-type prostate tissues (left) and intact FOXA1 Class 2 control and case tissues (right) (two-sided Wilcoxon test). J) Boxplots of distinct gene signature scores in intact FOXA1 Class 2 control and case tissues (top) and castrated or intact wild-type prostate tissues (bottom) (pseudo-bulk analyses from single-cell data, Wilcoxon test). K) Immunoblot of V5 and TROP2 in isogenic Class 2 Cre− (control) and Cre+ (Class 2 mutant expressing) mouse prostate organoids. Total H3 is used as a loading control. L) TROP2 immunofluorescence in Cre− and Cre+ Class 2 mutant mouse prostate organoids. Scale = 100μm. M) Left: Representative images from the limited dilution assay (100 cells/well) in Class 2 Cre− and Cre+ organoids. Right: Percentage of organoids formed in the limited dilution assay at varying cell numbers (n= 3 biological replicates, two-sided t-test). N) Reverse Kaplan-Meier plot of subcutaneous organoid grafting of wild-type, Class 1 or 2 monogenic mouse prostate organoids in *CB17/SCID* mice.

We engineered a knock-in mouse carrying the FOXA1 P358fs (Class 2) mutant transgene and, using a similar mating strategy with the Pb-Cre4 mice, conditionally overexpressed the mutant protein in the prostate luminal epithelia (fig. S7D). Despite robust expression, in contrast to Class 1 mutants, FOXA1 P358fs mutant did not trigger formation of precancerous PINs or hyperproliferative IDCs, even in 145-week-old animals (fig. S7E–H). However, given the cistromic dominance of Class 2 mutants(5) (Fig. 3A and fig. S7I–M), we investigated their downstream effects on chromatin accessibility and transcriptome. We profiled wild-type (control) and Class 2-mutant (case) prostate tissues using the single-cell multi-omics (gene expression + ATAC) assay. Louvain clustering of epithelial cells identified seven distinct clusters (fig. S8A), with clusters 3 and 4 predominantly comprising cells coming from the *FOXA1 P358fs+/−;Pb-Cre+* prostate tissues (Fig. 3B). These cellular clusters constituted over 35–40% of the Class 2-mutant epithelia and strongly expressed gene markers of secretory luminal (Lum) cells (Fig. 3C).While remaining negative for basal genes, Class 2 mutant-expressing cells in clusters 3 and 4 gained expression of genes specifically expressed in the luminal proximal (LumP) cells (Fig. 3C), which is a rare subpopulation of urethra-proximal epithelial cells possessing progenitor/stem-like properties and enhanced regenerative capacity (32). Based on this expression profile, we annotated cells in clusters 3 and 4 as FOXA1 Class 2-induced luminal stem-like cells (abbreviated as C2-iLum^Stem^).

To further confirm the hybrid secretory–proximal luminal character of C2-iLum^Stem^ cells, we defined a consensus stemness signature by intersecting gene markers of luminal progenitors (i.e., LumP, LumC, and L2) from three independent mouse prostate cellular atlases (32–34) (fig. S8B, see methods). Transcriptomic comparisons between C2-iLum^Stem^ and Lum populations from case and control tissues, revealed a striking up-regulation of most of the consensus signature genes (Fig. 3D), including well-characterized markers like *Tacstd2*, *Krt4*, and *Psca* (fig. S8C). Immunofluorescent staining of TROP2 (protein encoded by the *Tacstd2* gene) in Class 2-mutant prostate tissues corroborated a marked, 15–20-fold expansion of TROP2+ epithelial cells, which also stained for V5-tagged FOXA1 P358fs protein (Fig. 3E and fig. S8D). TROP2+/V5+ epithelial cells were uniformly distributed within distal and proximal regions in all prostatic lobes (Fig. 3E) and showed evident expansion even in prostatic glands harvested from 6-week-old mice (fig. S8E). Given their uniform spatial distribution, we next questioned if C2-iLum^Stem^ cells originated from the differentiated luminal compartment. Unlike the proximally located LumP cells, C2-iLum^Stem^ cells strongly expressed differentiated luminal markers like *Nkx3.1* and *Hoxb13* (Fig. 3F), and co-stained for AR, TROP2, and transgenic FOXA1 proteins (Fig. 3G). C2-iLum^Stem^ cells were also negative for LumP and basal cell identity markers *Ppp1r1b* and *Trp63*, respectively (Fig. 3F). Furthermore, we detected a significant increase in luminal progenitor signature in differentiated luminal cells from distinct lobes in Class 2-mutant prostate tissues (fig. S8F,G). Altogether, we found overexpression of the cistromically dominant FOXA1 P358fs Class 2 mutant to reprogram transcriptomes of differentiated luminal epithelial cells to activate progenitor/stemness gene programs—a phenomenon we herein refer to as induced intra-luminal plasticity.

Recent studies mapping the mouse prostate cellular atlas identified distinct sub-populations of luminal epithelial cells that persist after castration, which share in common expression of stemness genes, including *Tacstd2* (33–35). Consistently, we found a marked induction of TROP2 expression in the wild-type prostate epithelium after castration (fig. S9A). Through single-cell transcriptional profiling of residual tissues two weeks post-castration, we identified an adaptive gene program activated in AR+ epithelial cells. These castration-induced genes were also activated in the C2-iLum^Stem^ cell population of *FOXA1 P358fs+/*−*;Pb-Cre+* animals, notably even without androgen withdrawal (Fig. 3H, top; fig. S9B). A recent study defined a distinct subpopulation of luminal cells, called L1, that emerge 7 days post-castration and repopulate the epithelium upon androgen restoration (35). We found C2-iLum^Stem^ cells to highly express the L1 signature genes in androgen-replete tissues (Fig. 3H, bottom). Consistently, we detected a sustained expression of *Nkx3.1*—an AR up-regulated gene—in the C2-iLum^Stem^ cells, which was undetectable in the AR+ epithelial cells after castration (Fig. 3I). FOXA1 P358fs-expressing prostate tissues maintained comparable AR chromatin binding, co-factor enrichment, and transcriptional activity, while triggering activation of genes associated with luminal stemness and adaptation to castration (Fig. 3J, top, Fig. S9C,D). Contrastingly, castration induced the expression of analogous gene signatures in the prostate epithelium while simultaneously suppressing the AR pathway (Fig. 3J, bottom).

Overexpression of the FOXA1 P358fs mutant in prostate organoids also induced expression of TROP2, which was evident through *in situ* TROP2 staining in intact organoids (Fig. 3K,L, fig. S9E). This was also accompanied by a concomitant enrichment of the gene signature of luminal progenitor cells (fig. S9F). To phenotypically characterize the stemness character, we performed limited dilution assays. Here, Class 2-expressing prostate lines showed a 2–3-fold higher organoid-forming capacity relative to isogenic wild-type (Cre−) cells (Fig. 3M, fig. S9G,H). Moreover, Class 2-mutant prostate organoids were the only single-gene model to form palpable allografts when transplanted into mouse dorsal flanks, in contrast to both wild-type and Class 1-mutant organoids (Fig. 3N, fig. S9I). In summary, while incapable of driving transformation, FOXA1 Class 2 mutants activate progenitor and castration-adaptive gene programs in luminal epithelial cells in hormone-replete prostate tissues.

## Class 2 mutants activate KLF5/AP-1 neo-enhancers to drive castration resistance

**Fig. 4.**
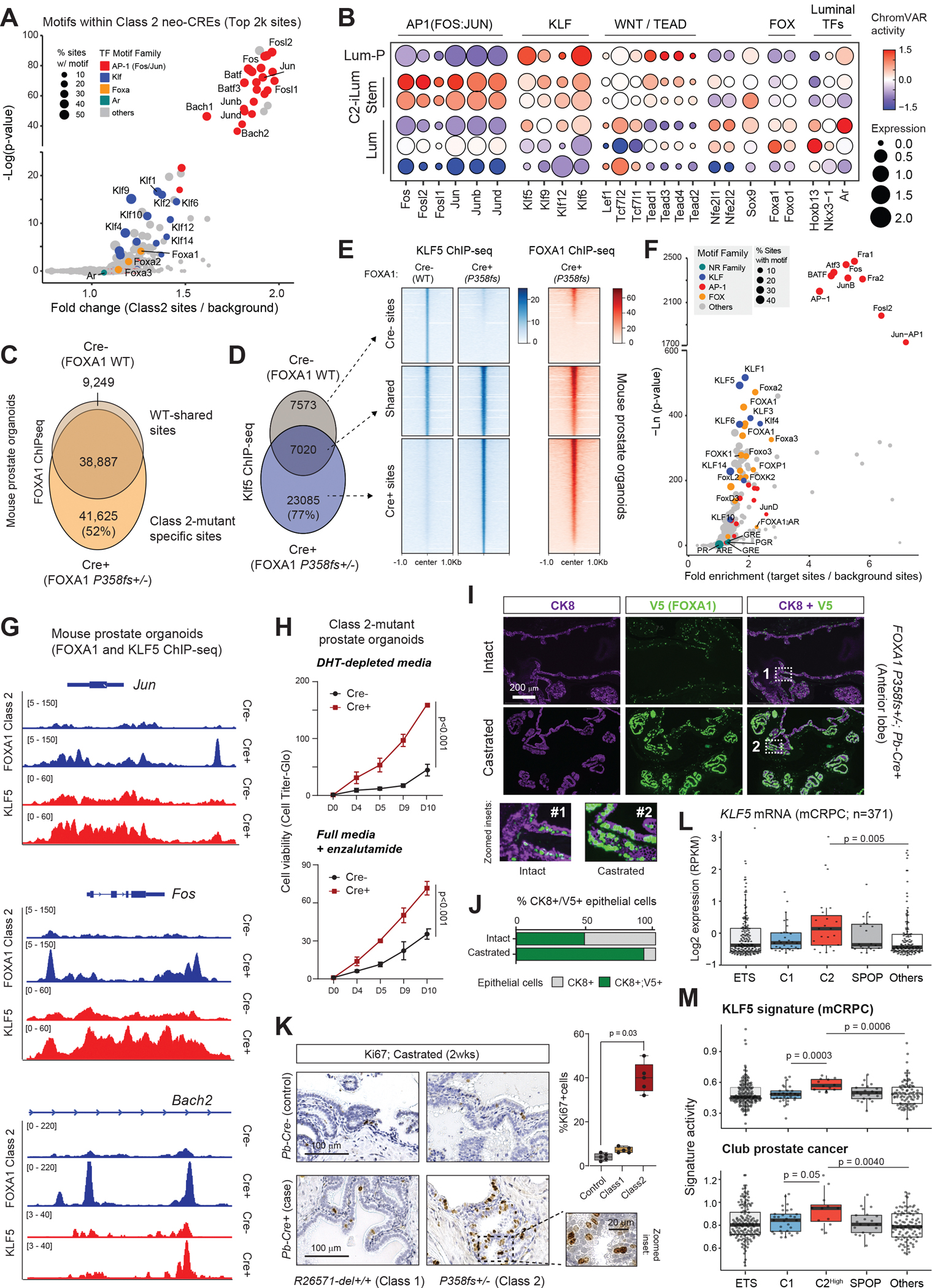
FOXA1 Class 2 mutants activate KLF5/AP-1 neo-enhancer circuitry to drive therapy-resistance. **A)** Fold change and significance of HOMER motifs enriched within Class 2 neo *cis*-regulatory elements (CREs) from single-cell multiome ATAC data in Class 2 control and case prostate tissues (HOMER, hypergeometric test). B) Bubble plots highlighting expression and chromVAR motif activity of noted transcription factors from 10X single-cell multi-omics of Class 2 mutant control and case tissues. The size of the dot indicates expression and heatmap color intensity indicates ChromVar activity. C) Venn diagram showing overlap of FOXA1 wild-type and Class 2 mutant cistromes in *P358fs+/*− Cre− and Cre+ mouse prostate organoids. D) Venn diagram showing overlap of the KLF5 cistrome in Class 2 Cre− and Cre+ mouse prostate organoids. E) ChIP-seq read-density heatmaps of KLF5 and FOXA1 Class 2 mutant at the Class 2-specific, wild-type, or shared sites in the FOXA1 *P358fs+/*− Cre− and Cre+ mouse prostate organoids. F) Fold change and significance of HOMER motifs enriched at KLF5 and Class 2 mutant co-bound sites (HOMER, hypergeometric test). G) ChIP-seq read-density tracks of the FOXA1 Class 2 mutant and KLF5 within the *Jun, Fos,* and *Bach2* locus in the Class 2 Cre− and Cre+ mouse prostate organoids. H) Growth curves (Cell Titer-Glow) of Cre− and Cre+ Class 2 mutant organoids in DHT-depleted and enzalutamide-treated media conditions (n=4 biological replicates, two-sided t-test). I) Representative multiplex-IF images of CK8 and V5 in the intact and castrated Class 2 mutant (*Pb-Cre+*) tissues. Scale =100μm. Zoomed insets of select regions are shown. J) Percentage of epithelial cells (CK8+, grey) that are V5+ (green) in intact and castrated Class 2 tissues from panel (I). K) Left: Representative Ki67 IHC stained sections of Class 1 (*R265–71del+/+*) and Class 2 (*P358fs+/*−) control and case castrated tissues. Right: Percentage of Ki67+ cells in control, Class 1, and Class 2 tissues (two-sided t-test). Box plot: center line, median; box, interquartile range (Q1–Q3); whiskers, minimum to maximum values; all individual data points shown. L) Boxplots showing *KLF5* mRNA expression in metastatic CRPC patient tissues (SU2C, n=371 patients) across distinct genomic driver groups. SU2C, Stand Up To Cancer (Wilcoxon rank-sum test). M) Boxplots showing the KLF5 gene signature (CRPC, top) and Club prostate cancer signature (bottom) in CRPC patient tissues (SU2C, n=371 patients) across distinct genomic driver groups (Wilcoxon rank-sum test).

Next, to interrogate the molecular mechanism of Class 2-mutant induced stemness, we compared chromatin accessibility profiles between C2-iLum^Stem^ and differentiated luminal cells using single-cell ATAC-seq data. Here, we detected a significant increase in open chromatin in the presence of the Class 2 mutant, with over 8,800 distal non-coding *cis*-regulatory sites gaining accessibility (fig. S10A). Motif analyses of Class 2-specific *cis*-regulome revealed a significant enrichment of motifs belonging to the AP-1 (FOS/JUN), KLF, and FOXA TF families (Fig. 4A), with a surprising absence of AR and other nuclear receptor motifs. To further elucidate differential activation of transcriptional networks across distinct prostate epithelial cell populations in Class 2-mutant tissues, we performed ChromVAR analysis using matched single-cell chromatin accessibility (ATAC) data. C2-iLum^Stem^ cells showed distinctive activation of AP-1 transcriptional complexes (Fig. 4B). KLF factors instead were predicted to have higher activity in both the innate LumP and C2-iLum^Stem^ clusters (Fig. 4B), with KLF5 showing higher expression in the C2-iLum^Stem^ cells relative to the differentiated luminal epithelium (fig. S10B). We also detected a significant activation of WNT (consistent with our earlier findings (5)) and TEAD (recently described to interact with AP-1 in a subset of CRPC tumors (36)) transcriptional regulators in C2-iLum^Stem^ epithelial cells (Fig. 4B).

Next, we employed our organoid models to molecularly characterize the crosstalk between FOXA1 P358fs mutant, AP-1, and KLF5 transcriptional complexes. FOXA1 ChIPseq revealed Class 2 mutant acquired *de novo* binding at over 40,000 new *cis*-regulatory sites within distal regions (Fig. 4C, fig. S10C,D). These Class 2-mutant-specific elements lacked the AR motif and instead showed enrichment of motifs recognized by AP-1 (FOS/JUN) family TFs (fig. S10E). As seen in mouse prostate tissues, overexpression of the FOXA1 P358fs mutant in prostate organoids also led to an up-regulation of KLF5 (fig. S10F), with the Class 2 mutant protein occupying *cis*-regulatory sites within the *Klf5* gene locus (fig. S10G). Even the endogenous FOXA1 P358fs mutant in LAPC4—a human-derived CRPC cell line—showed robust binding within the *Klf5* gene locus (fig. S10G).

KLF5 was recently implicated in driving castration resistance in human PCa (37). Thus, we next profiled KLF5 chromatin occupancy (via ChIP-seq) in our Class 2-mutant organoids. KLF5 gained binding at over 23,000 new sites (Fig. 4D) in presence of the FOXA1 P358fs mutant, which was co-occupied by the FOXA1 P358fs mutant (Fig. 4E). HOMER motif analysis of Class 2 and KLF5 shared enhancers uncovered a significant enrichment of their own as well as the AP-1 family TF motifs (Fig. 4F). We also found FOXA1 P358fs to bind within gene loci of several AP-1 family TFs, including *Jun*, *Fos*, and *Maff,* which was accompanied by a concurrent gain in KLF5 binding at these sites and up-regulation of their transcripts (Fig. 4G, fig. S10H,I).

In phenotypic assays, we found Class 2-mutant prostate organoids to grow significantly faster in androgen-depleted media as well as upon treatment with the AR antagonist enzalutamide (Fig. 4H). To assess this *in vivo*, we castrated the *FOXA1 P358fs+/−;Pb-Cre+* mice and quantified the survival of transgene-expressing luminal epithelial cells. Here, we focused on the anterior prostate lobes leveraging the well-described incomplete Cre recombinase activity that leads to transgene expression in about half of the epithelial cells (25). Accordingly, in intact animals, we detected about 50% of the CK8+ luminal epithelial cells to co-express the V5-tagged FOXA1 P358fs transgenic protein (Fig. 4I,J). CK8+;V5+ epithelial cells, however, comprised almost 95% of the prostate epithelium two weeks post-castration (Fig. 4I,J), suggesting that FOXA1 P358fs-expressing cells can survive and persist upon androgen removal. Consistently, Ki67 staining of residual prostate tissues revealed that the Class 2-mutant epithelia had significantly higher proliferative activity in the absence of androgen relative to both wild-type and Class 1-mutant cells (Fig. 4K). This sustained proliferative capacity was also reflected in significantly higher weights of residual prostate glands harvested from the case animals (fig. S11A).

Finally, in human patient tumors, we found the highest expression of KLF5 in FOXA1 Class 2-mutant CRPC specimens (Fig. 4L). In Class 2-mutant tumors, KLF5 expression was also positively correlated to two independent gene signatures of KLF5 (fig. S11B; published in (37)). Assessment of KLF5 activity based on both signatures also uncovered strongest activation in Class 2-mutant CRPC tumors (Fig. 4M, fig. S11C). In Class 2-mutant tumors, we also detected a strong activation of gene signature of human PCa-associated club cells (Fig. 4M), which were identified via single-cell methodologies in metastatic CRPC specimens (38) and are associated with treatment resistance to AR-targeted therapies (39). Furthermore, we observed a strong positive correlation between *KLF5* transcripts and the PCa club signature in CRPC tumors, which showed no association in Class 1-mutant tumors (fig. S11D). Altogether, our findings suggest that FOXA1 Class 2 mutants reshape the *cis*-regulatory chromatin landscape in luminal epithelial cells to activate KLF5 and AP-1 enhancer-driven gene programs that confer resistance to ADT, positioning acquired Class 2 mutations as promoters of metastatic and therapy-resistant disease progression.

## Discussion

Using transgenic mouse models, we show that FOXA1 Class 1 mutations trigger formation of invasive adenocarcinomas, while Class 2 mutations rewire the luminal cell identity to impart resistance to ADT. Since the clinical demonstration of tumor regression following androgen blockade, inhibition of the androgen/AR axis has remained the cornerstone of PCa treatment (12, 40). However, replicating acute androgen dependence of primary PCa had been particularly challenging in mouse models. The widely-used PTEN inactivation-driven PCa mouse models typically result in prostatic tumors exhibiting castration-resistant phenotypes (41), thus representing a more advanced disease. Here, we found Class 1-driven prostate adenocarcinomas faithfully recapitulate key features of primary human PCa, including showing robust AR expression and sensitivity to androgen deprivation upon castration. Thus, these models could serve as valuable preclinical systems for investigating mechanisms underlying PCa initiation and development of castration resistance.

Mechanistically, Class 1 mutants simultaneously activate AR and PI3K/mTORC1 growth pathways. Similar transcriptional changes were observed in the recently developed SPOP-driven PCa murine model (42). Also, localized human PCa harboring either SPOP or FOXA1 Class 1 mutations exhibited elevated AR activity (5, 42). These insights are particularly significant given the well-established antagonistic relationship between AR and PI3K pathways in PCa (43), and lower prevalence of PI3K pathway alterations in the organ-confined, localized disease (2, 3, 44). In this context, we posit that Class 1 mutations amplify AR activity to counteract the inherent antagonism of AR signaling resulting from non-canonical activation of oncogenic mTORC1 signaling in these tumors. Our findings also offer a plausible explanation for lower frequency of PTEN alterations during metastatic progression of FOXA1 Class 1-initiated primary tumors.

In contrast to Class 1 mutations, C-terminal truncating Class 2 mutations failed to initiate PCa formation. This is consistent with clonal presentation of Class 2 mutations only in CRPC tumors (5). Class 2 mutants instead triggered profound reprogramming of the chromatin regulatory landscape by commissioning a KLF5/AP-1 neo-cistrome in prostate luminal epithelial cells that specifies a TROP2+/CK4+ progenitor cell state and activates castration-induced gene programs even without exposure to ADT. This suggests that Class 2 mutants serve as promoters of PCa progression by “priming” the prostate epithelium to resist ADT. Earlier, we had shown Class 2 mutants promote metastasis in human cancer models by de-repressing the WNT pathway (5). Thus, we propose a coalescent model wherein Class 2 mutations emerge as primary prostatic tumors subsequently evolve towards metastatic (via WNT de-repression) or castration-resistant (via inducing intra-luminal plasticity) phenotypes. However, further research is needed to determine if FOXA1 Class 2 mutations emerge spontaneously during natural disease progression—such as in *de novo* metastatic tumors—or are instead induced by the selective pressure of androgen/AR blockade.

Enrichment of AP-1 TF motifs in the Class 2 neo-cistrome is consistent with emerging literature implicating this complex in driving therapy-emergent PCa subtypes (36), including AR-negative neuroendocrine disease (45). A comprehensive mouse prostate cellular atlas by Tang and colleagues recently identified a castration-induced luminal cell population, designated L1, which persists following castration and repopulates the epithelial lining upon androgen restoration (35). This study suggested this luminal plasticity to be regulated by the loss of AR and gain of AP-1 *cis*-regulatory elements. In this study, while maintaining AR activity, we found Class 2 mutants to parallelly co-opt AP-1 and KLF5 enhancers for driving luminal plasticity in androgen-replete mouse prostate epithelium. Our analyses also revealed increased activity of TEAD family factors in the Class 2 mutant-specific *cis*-regulome. Recent studies using human patient specimens reported TEAD proteins to collaborate with AP-1 in establishing a distinct chromatin profile in stem cell-like CRPC tumors (designated as CRPC-SCL) that show shorter remission upon treatment with AR signaling inhibitors (36). These insights position AP-1 as key co-factors in driving lineage plasticity—both intra-luminal as well as neuroendocrine—that enables PCa progression and resistance to androgen/AR-targeted therapies.

**Fig. 5.**
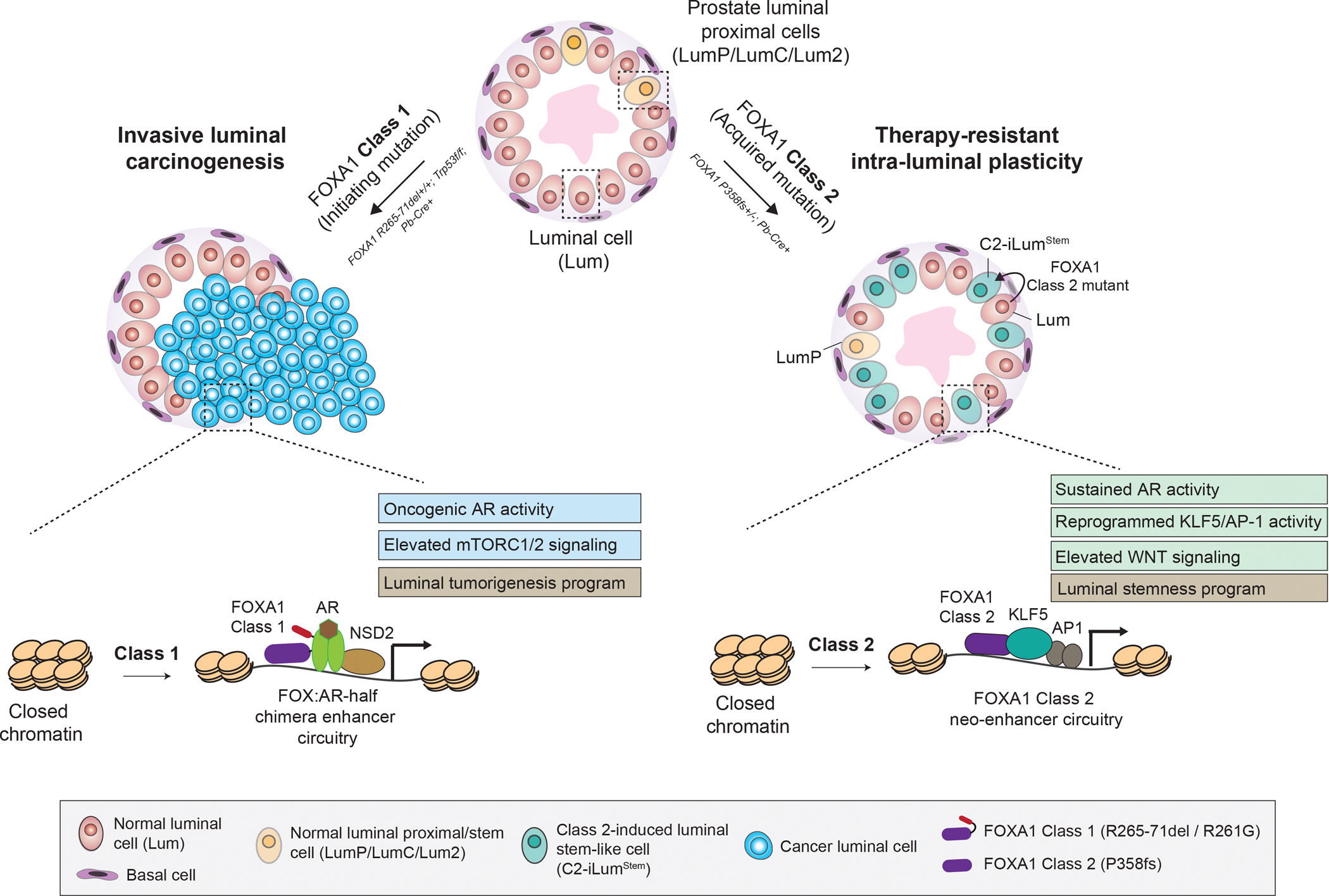
Schema depicting distinct oncogenic mechanisms of FOXA1 Class 1 and Class 2 mutations in driving prostate tumorigenesis or therapy-resistant progression. Normal prostate epithelia comprise three main cell types: terminally differentiated, secretory luminal (Lum) cells, basal cells, and luminal proximal stem-like cells (annotated as LumP, LumC, or Lum2 in published cellular atlases). Overexpression of FOXA1 Class 1 mutants in a p53-deficient prostate epithelium (left) drives formation of androgen-sensitive, invasive prostate adenocarcinoma. This malignant transformation is enabled by the aberrant induction of NSD2, cistromic redistribution of the androgen receptor (AR) at chimeric FOXA1:AR-half enhancer elements, and co-activation of the mTORC1/2 oncogenic pathway. In contrast, overexpression of FOXA1 Class 2 mutants (right) reprograms AR+/CK8+ differentiated luminal epithelial cells to express luminal stemness markers, thereby partially acquiring progenitor-like characteristics. Class 2 mutants induce this intra-luminal plasticity by commissioning a *neo*-cistrome that is occupied and activated by the AP-1 and KLF5 transcriptional complexes. Class 2-induced luminal stem-like cells (annotated C2-iLum^Stem^) retain AR activity and resist cell death upon castration, suggesting their role in enabling PCa progression following androgen deprivation therapy.

In summary, our study provides the first experimental evidence that distinct classes of FOXA1 alterations drive disparate PCa phenotypes (Fig. 5), while explaining the divergent evolution of Class 1 and Class 2 mutations in human disease. Further validating the originally proposed structural classification schema of FOXA1 alterations (5, 6), these findings underscore functional versatility of FOXA1 as a principal PCa oncogene. Depending on the mutation type, FOXA1 either drives PCa formation or metastatic, therapy-resistant progression. Furthermore, FOXA1 transgenic mouse models developed in this study provide a valuable bioresource for studying the pathobiology of breast, salivary gland, and bladder tumors, which harbor recurrent FOXA1 alterations.

## Materials and Methods

### Ethical statement

All experiments detailed in this paper complied with the Institutional Review Board and the Institutional Animal Care and Use Committee (IACUC) at the University of Michigan.

### Animal procurement

Animal studies were approved by the Institutional Animal Care and Use Committee at the University of Michigan. Animal use and care were in strict compliance with institutional guidelines, and all experiments were performed in compliance with relevant regulatory standards laid down by the university. Transgenic FOXA1 mouse lines were obtained from Baylor College of Medicine and were generated in a *C57BL/6* background. *Probasin-Cre* (Stock#026662) mice and *Trp53f/f* (Stock#008462) mice were obtained from Jackson Laboratory (Bar Harbor, ME). For the generation of the syngeneic models, male *C57BL/6J* (Stock# 000664) mice (6–8-weeks old) and NOD Cg-Prkdc<scid> ll2rg<tm1Wjl>SzJ (NSG) mice were obtained from the Jackson Laboratory (Stock#005557). For the subcutaneous grafting assay, male *CB17* SCID mice were obtained from Charles River (Stock#236). Mice were housed in a pathogen-free animal barrier facility under a pathogen-free, 12 h light/12 h dark cycle, 18–23 °C temperatures, and 40–60% humidity.

### Generation of FOXA1 transgenic mice

FOXA1 mutant transgenic models were generated as described earlier (41). Briefly, plasmids containing CMV enhancer-driven human FOXA1 mutations (Class 1, 2, and 3) were injected into the pronuclei of *C57BL/6* mouse fertilized eggs, which were then implanted into pseudo-pregnant females. While these models were generated using the human FOXA1 coding sequence, the mouse and human FOXA1 sequences are highly conserved, with a 96.6% similarity at the amino acid level, with the Wing 2 and forkhead functional domains being identical. These experiments were performed as a service in Dr. Jianming Xu’s laboratory at Baylor College of Medicine, Houston, Texas. PCR screening was used to identify potential founder animals. Sperms from all transgenic lines have been cryopreserved at the University of Michigan and can be revived and shared with the scientific community upon request. Genotyping of tail DNA from FOXA1 mutant mice was performed using Agarose Gel Electrophoresis and PCR. The following sequences of primers were used for PCR analysis:

Genotyping_Left_F: 5′-GAGTTCTCTGCTGCCTCCTGG-3′

Genotyping_Left_R: 5′-GAGCGGCCAGCTTATCGATAC-3′

Genotyping_Right_F: 5′-GAAAGAACCAGCTGGGGCTC-3′

Genotyping_Right_R: 5′-CGAAAATCTGTGGGAAGTCTTG-3′

Span_Forward: 5′-GAGTTCTCTGCTGCCTCCTGG-3′

Span_Reverse: 5′-CGAAAATCTGTGGGAAGTCTTG-3′

The Genotyping Left (GT-L) primers generated a product size of 225 bp, and the Genotyping Right (GT-R) primers generated a product size of 385 bp, while the span primers generated a product size of 213 bp. PCR conditions used were 35 cycles (15 s at 94 °C, 30 s at 58 °C and 30 s at 72 °C) using high fidelity Platinum^™^ Taq DNA Polymerase (Thermo Fisher Scientific, Cat#11304011), 10 μM of the primers and 2μl of the tail DNA. For the castration experiments, sham or castration surgeries were performed in compliance with the University of Michigan IACUC guidelines.

### Allograft model

Organoids were trypsinized, filtered, and resuspended in Matrigel for subcutaneous injections. Briefly, 3 × 10^6^ cells were injected into the dorsal flanks of NSG mice. Tumor growth was monitored weekly. Once *R265–71del+/+; Trp53f/f* tumors reached 1000 mm^3^, the resected tumors were cut into small size pieces and implanted into the subcutaneous space of both flanks of *C57BL/6* mice to generate the syngeneic model.

### Antibodies

For immunoblotting, the following antibodies were used: AR (Millipore: 06–680); V5 tag (Thermo Fisher Scientific: R960–25); FOXA1 (Millipore: 05–1466); c-MYC (Cell Signaling Technologies: 5605S); vinculin (Sigma Aldrich: V9131); H3 (Cell Signaling Technologies: 3638S); KLF5 (Proteintech: 21017–1-AP); phospho-AKT (Ser473) (D9E) (Cell Signaling Technologies: 4060S); phospho-S6 ribosomal protein (Ser235/236) (Cell Signaling Technologies: 2211S).

For immunohistochemistry, the following antibodies were used: Ki67 (BD Pharmingen^™^: 550609); V5 tag (Thermo Fisher Scientific: R960–25); AR (Millipore: 06–680); FOXA1 (Thermo Fisher Scientific: PA5–27157); TROP2 (Bio-Techne R&D Systems: BAF1122); alpha-SMA (Ventana Medical Systems, 760–2833).

For immunofluorescence, the following antibodies were used: CK8 (Abcam: ab53280); AR (Ventana: 760–4605, Abcam: ab108341); V5 tag (Thermo Fisher Scientific: R960–25); TROP2 (Bio-Techne R&D Systems: BAF1122); NSD2 (Abcam: ab75359); NKX3.1 (Athena Enzyme Systems/Athena Environmental Sciences 0317).

For ChIP-seq, the following antibodies were used: AR (Millipore: 06–680); KLF5 (Proteintech: 21017–1-AP); FOXA1 (Thermo Fisher Scientific: PA5–27157); FOXA1 (Cell Signaling Technology: 53528).

### Immunoblotting

Cell lysates were prepared and immunoblotting was performed as described earlier (46). Briefly, cells were lysed in RIPA buffer (ThermoFisher Scientific: 89900) containing Protease and Phosphatase Inhibitor Cocktail (ThermoFisher Scientific: 78440) and denatured for 10 minutes by heating at 70 °C. Next, Pierce 660nM protein assay reagent was used to measure the protein concentration (Thermo Fisher Scientific: 22660). 10–30 μg of total protein was loaded per lane and separated in either NuPAGE 3 – 8%, Tris-Acetate Protein Gel (ThermoFisher Scientific) or NuPAGE 4 – 12%, Bis-Tris Protein Gel (ThermoFisher Scientific), and transferred in semi-dry transfer system (Trans-blot Turbo System; BioRad) at 25 volts for one hour. Membrane blocking was performed for one hour in 5% non-fat dry milk and incubated overnight at 4°C with primary antibodies. Secondary antibodies with horseradish peroxidase (HRP; BioRad) conjugation were used at 1:20,000 diluted in TBST, and the membrane was incubated for one hour at room temperature. Blots were finally developed using enhanced chemiluminescence (ECL Prime, Thermo Fisher Scientific) and imaged on an Odyssey Fx Imager (LiCOR Biosciences) following the manufacturer’s protocol.

The following antibodies were used for immunoblotting: AR (Millipore: 06–680); V5 tag (Thermo Fisher Scientific: R960–25); FOXA1 (Millipore: 05–1466, Thermo Fisher Scientific: PA5–27157); c-MYC (Cell Signaling Technologies: 5605S); vinculin (Sigma Aldrich: V9131); H3 (Cell Signaling Technologies: 3638S); KLF5 (Proteintech: 21017–1-AP); PTEN (D4.3) (Cell Signaling Technology: 9188S); AKT (Cell Signaling Technology: 9272S); phospho-AKT (Ser473) (D9E) (Cell Signaling Technologies: 4060S); S6 ribosomal protein (5G10) (Cell Signaling Technologies: 2217S); phospho-S6 ribosomal protein (Ser240/244) (Cell Signaling Technologies: 2215S), TROP2 (Thermo Fisher Scientific: PA5–86376).

### Generation of mouse prostate organoids

To generate mouse prostate organoids, mice were euthanized, and the distinct prostate lobes were micro-dissected. Unless specified otherwise, all four prostate lobes (anterior, dorsal, ventral, and lateral) were pooled and minced using a disposable scalpel into small pieces of approximately 1.0 mm3 in a 10-cm culture dish under aseptic conditions. Subsequently, the minced tissues were transferred into 15 mL tubes containing prewarmed (37 °C) advanced DMEM F-12 +/+/+ media with 5 mg/mL of collagenase Type II (Gibco, 17101015) and incubated on a rotator for 1 hour at 37°C. Next, the tissues were digested with TrypLE (Thermo Fisher Scientific, 12605010) for 15 minutes, followed by a media wash. The cell pellets were finally suspended in 90% growth factor reduced Matrigel (Corning^®^, 354230) diluted with advanced DMEM F-12 media on ice and plated in a 24-well tissue culture plate (40 μL/drop). After solidifying the gel at 37 °C in the 5% CO2 incubator for 15 minutes, 0.5 mL/well of complete mouse prostate organoid culture media were added. The components of the mouse prostate media were adapted from previously published protocols(47).

### Organoid Adenoviral Cre and CRISPR/Cas-9 lentiviral transduction

*In vitro* CRE recombination was achieved by adenoviral delivery of CRE recombinase-containing virus or empty vector based on previously published protocols (48). For the adenoviral transduction, 5μl of a 10X viral stock was added to 100k cells, along with 16μg/mL polybrene, and incubated for 1 hour at 37°C on a rotor. Following this, the tubes were centrifuged for one hour at 750×g at 25°C, washed twice with 1X PBS, and plated into four 40μl Matrigel domes. Immunoblots for V5 were run to confirm recombination and transgene expression.

Similarly, CRISPR/Cas-9 mediated knockout of *Trp53* and *Nsd2* was performed by lentiviral delivery of a CRISPR-V2 plasmid encoding an active Cas9 and sgRNA sequences. Briefly, 1 mL of the 10x lentivirus was added along with polybrene to 100k cells. Spinfection was performed as described above, and immunoblotting or immunohistochemistry was performed to validate protein knock-out. The sgRNAs used in this study are as follows:

sg*Trp53* #1:5’-CACCGGCGGTTCATGCCCCCCATGC-3’

sg*Trp53* #2:5’-CACCGGTGAAATACTCTCCATCAA G-3’

*sgNsd2*#1:5’-CACCGTTGATAGGTGTAGTATTGGG-3’

*sgNsd2*#2:5’-CACCGCCTTAGCTACTTGAGGGTTG-3’

### Organoid growth and limiting dilution assays

Prior to plating for growth assays, organoids were digested with TrypLE for 10 minutes. 250–500 cells were plated per Matrigel dome in a 12-well plate in differing media conditions. Cell viability was assayed at Day 0 for six days according to the CellTiter-Glo 3D Cell Viability Assay Kit (Promega G9683).

For the organoid limited dilution assay, 50, 100, 500, and 1000 cells were plated in 40μl or 25μl Matrigel domes in a 24-well plate (manual) or 96-well plate (IncuCyte), respectively, in complete mouse prostate organoid media. For the manual assay, organoids were cultured for eight days before imaging, while for the IncuCyte, images were captured every day, and representative images from Day 8 were shown.

### *In situ* proximity ligation assay (PLA)

PLA was performed using a previously described protocol (49). Briefly, FOXA1 Class 1 mutant NSD2 wild-type and NSD2 knock-out organoids were fixed for 10 minutes in 4% PFA and washed thrice with 1x PBS. After ethanol-based dehydration and sectioning, slides were deparaffinized and antigen retrieval was performed by boiling slides for 15 minutes in citrate buffer (pH 6.0). Next, slides were permeabilized using 0.5% Triton X-100% (in PBS) for 10 min. The NaveniFlex PLA kit staining kit was used with mild modifications. Briefly, slides were blocked in 10% goat serum, followed by incubation with the desired primary antibodies (AR abcam, Cat#ab108341; NSD2 abcam, Cat#ab75359) at four degrees overnight. Finally, slides were mounted using Prolong Gold Antifade mounting media and the PLA signal intensity/spot was determined with ImageJ software.

### RNA isolation and sequencing

Prostate organoids were seeded at a density of 20,000 cells/40μl Matrigel dome in 2 replicates on Day 0 and incubated for 48 hours in complete organoid media, followed by a 12-hour starvation in media lacking EGF and DHT. Organoids were stimulated with 10 nM DHT for 24 hours to define a mouse-specific AR signature. RNA isolation and library preparation were performed using previously described protocols(5, 49). Briefly, total RNA was then isolated using QIAzol Lysis Reagent (QIAGEN) and extracted using the miRNeasy Mini Kit (Qiagen). SuperScript III Reverse Transcriptase enzyme (Thermo Fisher Scientific) was used for conversion of 1ug RNA into cDNA following the manufacturer’s instructions.

RiboErase RNA-seq libraries were prepared using 200–1,000 ng of total RNA extracted from mouse organoids. Ribosomal RNA was eliminated through enzymatic digestion using the KAPA RNA Hyper+RiboErase HMR Kit (Roche). RNA was then fragmented to approximately 200–300 bp using heat and double-stranded cDNA was synthesized after fragmentation, followed by end-repair and ligation using New England Biolabs adapters. The final library preparation involved amplification (2x KAPA HiFi HotStart mix) and addition of NEB dual barcodes, following the manufacturer’s protocol. Quality control of the libraries were performed using the Agilent 2100 Bioanalyzer and paired-end sequencing was performed on the Illumina Novaseq, with 2 × 100 nucleotide read lengths and a sequence coverage of 15–20 million paired reads per sample.

Alignment was performed using Kallisto (v0.46.1) to the mouse (mm10/GRCm38) or human (hg38/GRCh38) references(50). Read counts were adjusted for normalization and filtered in R via package EdgeR (51). Package Limma-Voom (vlimma_3.53.10) was involved in differential expression analysis (52). GSEA analysis and corresponding heatmaps and figures were created using R package fgsea (vfgsea_1.24.0), ComplexHeatmap, and ggplot2 for signatures from MSigDB’s hallmark MTORC1 and custom AR signatures based on our data (53–55).

### ChIP-seq and analyses

Prostate organoids were seeded at a density of 30,000 cells/well and grown for 3–5 days in complete media. Organoids were stimulated with 1 nM of R1881 for 6 hours before harvest. Organoids were dissociated using TrypleLE for 10 minutes and split into 500,000 to 1 million cells per tube.

Chromatin immunoprecipitation (ChIP) experiments were performed as described earlier(5, 56). The Ideal ChIP-seq Kit for transcription factors (Diagenode) was used following the manufacturer’s protocol. 500,000 to 1 million cells were used per ChIP reaction with 2μg of antibody. Briefly, cells were crosslinked, lysed and sonicated using a Bioruptor Pico, Diagenode machine to achieve a desired chromatin size of about 200bp. Sheared chromatin was incubated with desired antibodies overnight at 4 °C followed by de-crosslinking and DNA purification the next day.

Purified DNA was prepared for sequencing using the manufacturer’s instructions (Illumina). Library preparation and processing of reads were performed as described earlier (56). Quality control of the final libraries were performed using the Bioanalyzer 2100 (Agilent) and sequenced on the Illumina Novaseq Sequencer (125-nucleotide read length).

Reads were processed as described in Parolia et al (10). Briefly, reads were trimmed using Trimmomatic version 0.39 (settings TruSeq3-PE-2.fa:2:30:10, minlen 50), and aligned using bwa (“bwa mem,” options −5SP -T0, version 0.7.17-r1198-dirty) to mm10 (GRCm38) genome reference or hg38 (GRCh38) reference(57, 58). Next, reads were filtered using Markduplicates from Picard in addition to a quality score filtering of >20 via samtools(59). Peak calling was performed using MACS2, filtered using bedtools, and converted to bigwigs with UCSC wigtoBigwig(60, 61). Cistrome overlap analysis was performed in R (v3.6.0) using ChipSeekAnno (v3.0.0) and ChipSeeker (v1.29.1)(62, 63). Enrichment heatmaps were generated using Deeptools(64). HOMER was used to perform motif enrichment analysis (65). Motif dot plot summaries were created in R (v3.6.0) using Sushi (66).

### Single-cell RNA and multi-omics (ATAC+RNA) sample preparation

Mice were euthanized, and distinct prostate lobes were micro-dissected. Unless specified otherwise, all four prostate lobes were pooled and minced using a disposable scalpel into small pieces of approximately 1.0 mm^3^ in a 10-cm culture dish under aseptic conditions. Subsequently, the minced tissues were transferred into 15 mL tubes containing prewarmed (37 °C) advanced DMEM F-12 +/+/+ media with 5 mg/mL collagenase Type II (Gibco, 17101015) and incubated on a rotator for 30 minutes at 37°C. Next, the tissues were digested with TrypLE (Thermo Fisher Scientific, 12605010) for 15 minutes, followed by a media wash. Single cells were strained using a 70 and 40-micron filter, followed by a 0.4% BSA in 1XPBS wash. Following this, cells were either processed for single-cell RNA-sequencing or 10x single-cell multi-omics (ATAC+RNAseq).

Single-cell RNA sequencing was performed using the Chromium Next GEM Single Cell 3’ Kit v3.1 (10x Genomics) according to the manufacturer’s instructions (67). Briefly, single-cell suspensions were loaded onto a Chromium Next GEM Chip along with reverse transcription reagents and Gel Beads containing barcoded oligonucleotides. The chip was then placed in a Chromium Controller to generate Gel Bead-in-Emulsions (GEMs), encapsulating individual cells with unique barcoded Gel Beads. Cellular mRNAs were reverse transcribed within each GEM to generate barcoded full-length cDNA. Following reverse transcription, the GEMs were broken, and the barcoded cDNA was amplified by PCR. The amplified cDNA underwent fragmentation, end repair, and A-tailing, followed by adapter ligation and PCR amplification to generate final sequencing libraries. Library quality was assessed using an Agilent Bioanalyzer.

Single-cell multi-omics was performed using the Chromium Next GEM Single-cell Multiome ATAC+Gene expression kit (10x Genomics) according to the manufacturer’s protocol (68). Briefly, nuclei were isolated, followed by transposition and adapter ligation using a Tn5 transposase. Transposed nuclei were loaded onto a Chromium Next GEM microfluidic chip along with gel beads containing 10x barcoded oligonucleotides. The GEMs were incubated to produce barcoded DNA from transposed chromatin (ATAC) and barcoded full-length cDNA from polyadenylated mRNA (Gene expression) within each GEM. Following this, the GEM was broken, cDNA was amplified, and pre-amplified cDNA was used as input for the ATAC and gene expression library preparation. For ATAC, sample indexing primers were used for further amplification while the amplified cDNA underwent fragmentation, end repair, and A-tailing, followed by adapter ligation and PCR amplification to generate final sequencing libraries. Library quality was assessed using an Agilent Bioanalyzer.

### Single-cell RNA-seq and multiome (single nucleus RNA-seq and ATAC-seq) sequencing

*Single-cell RNA-sequencing:* Data processing was performed using a previously described protocol (49). Briefly, after sequencing, read demultiplexing, alignment, and gene quantification were conducted with the 10X Genomics Cell Ranger pipeline (v5.0) for scRNA libraries or Cell Ranger ARC pipeline (v2.0) for multiome libraries. Custom reference was built by adding sequences of human FOXA1 and puroR to the prebuilt mouse reference (mm10) downloaded from the 10X website. To reduce potential ambient RNA contamination, especially for nuclear libraries, SoupX (69) was applied to the raw gene count matrix obtained from Cell Ranger, and the corrected read count matrix was used for downstream analyses with Seurat (v4.1)(70) if not specified otherwise. Using the median number of detected genes and fraction of mitochondrial reads per cell, low-quality cells were further filtered (table S3). In addition, putative doublets were identified using the pre-SoupX matrix with scDblFinder (71) followed by removal of mitochondrial genes from the matrix. After QC steps, the SoupX corrected count matrices of replicates from paired libraries (e.g., case and control) were pooled and normalized. The top 2000 highly variable genes were identified followed by assignment of cells to clusters. Cell annotation was performed using the TransferData method and public datasets as reference (32–34), which were manually inspected and adjusted according to top marker gene expression. Sub-clustering was conducted with all cells annotated as Basal, Luminal, or LumP to capture the heterogeneity of epithelial cells.

To conduct gene set enrichment analysis (GSEA) between groups, pseudo-bulk gene expression profiles were generated and TPM-based normalized expression was calculated with edgeR by using the TMM scaling factors (51). In Class 1 case versus control comparison, luminal cells in case that were puroR−;hFOXA1+ (based on imputed data) were compared to luminal cells in control that were puroR+; whereas for basal cells, puroR+ were used for case and control. Genes ranked by logFC were used as input for preranked GSEA with fgsea (72). MsigDB was used to download Hallmark gene sets (73). When noted, MAGIC (74) imputed gene expression was used for data visualization with heatmap and violin plot. Pseudo-time analysis was performed with Slingshot (75) using MAGIC imputed gene expression matrix. The gene expression analyses of the multiome libraries followed the same pipeline as the single-cell RNA-seq libraries described above. Prediction of cell cycle position was performed with tricycle (76)

*Gene signatures:* Gene signatures from Class 1 and Class 2 single-cell RNA-sequencing or multi-omics data were defined by performing pseudo-bulk analyses between the case and control-specific clusters. Pure tumor cells in the pseudo-bulk analyses of 100-week-old single-cell RNA-sequencing data were identified by expression of transgenic FOXA1 and lack of puroR mRNA expression. This selection strategy enabled the removal of cells within case tissues that escaped Cre recombination, thus retaining puroR transcript expression.

The AR signature (only comprising activated genes) was defined using common genes up-regulated in wild-type mouse prostate organoids stimulated with DHT (10 nM) for 24 hours. mTOR signature from the MSigDB hallmark database (HALLMARK_MTORC1_SIGNALING) was used. Basal, Lum, and LumP signatures were selected from the top 25 up-regulated genes in differential analysis between one versus others (ranked by p values from Wilcoxon’s test) using the single-cell data from a 100-week control mouse. Top marker genes of basal cells expressed in LumP cells were excluded to avoid overlapping signatures. Prostate adenocarcinoma-related transcription factors were obtained from a previous publication (26). The FOXA1-driven PCa signature was defined as the top 50 up-regulated genes in the Class 1 case vs control luminal cell clusters (fig. S3F). Markers for LumP (32–34),Luminal-2 (32–34), and Luminal-C (32–34) were identified using data and annotation from corresponding studies by comparing to all other epithelial cells. Consensus stemness (42 genes) signature was determined from the intersection of the top 100 up-regulated genes in LumP, Luminal-2, and Luminal-C (fig. S8B).

The castration signature was derived using multiome gene expression data collected from wild-type prostates or residual prostate tissues 2 weeks post-castration. Specifically, differential analysis was conducted between castrated and wild-type (i.e., hormonally intact) epithelial cells at the pseudo-bulk level, and the top 20 up-regulated genes were identified. Genes with similar expression levels in basal cells were filtered out (the difference in mean expression between castrated luminal and basal was <1 log2TPM).

*Single-cell ATAC-seq:* The ATAC-seq data of the multiome libraries from the Class 2 case and control animals were pooled and analyzed together. To filter low-quality cells, only cell barcodes associated with more than 1000 fragments in peaks were kept. If not specified otherwise, all downstream analyses were performed with Signac (v1.5.0)(77) and Seurat (v4.1)(70). To integrate different libraries, a common set of peak regions was first created by merging peak regions of all libraries; fragments in the consensus peak set were then re-counted using the raw fragments data for each library. The resultant peak cell matrices from all libraries were pooled and subjected to term frequency-inverse document frequency (TF-IDF) normalization. Subsequently, linear dimension reduction data was performed based on the top 50% abundant peaks using singular value decomposition (SVD). The top 30 latent semantic indexing (LSI) components, except the first component, were used to perform nonlinear dimension reduction using UMAP and construct a shared nearest neighbor graph before clustering with the SLM algorithm. To find differentially accessible regions (DARs) between cell groups, the findMarker function was utilized with the LR (logistic regression) test method and the total number of fragments as a latent variable. Motif enrichment in the top 2000 DARs was calculated using a hypergeometric test. Motif activity score at the cell level was calculated by running chromVAR (78) with JASPAR2020 motifs (79). biomaRT was used to map the mouse homolog genes of the human signature (80).

### Cohorts, datasets, and resources

Pie charts for FOXA1 mutations were made from previous studies (2, 3, 5, 6, 8, 81).

For the TCGA prostate adenocarcinoma RNA-seq analysis, the TPM matrix was downloaded from UCSC Xena browser (https://xena.ucsc.edu/)(82). FOXA1 class 1 cases were identified using an in-house pipeline (83). PI3K altered cases include cases with driver mutations in AKT1, PIK3CA, PTEN or BRAF and cases with PTEN homozygous deletion; cases with ETS fusions were excluded. Cases that are not grouped into the major molecular subtypes (ETS, SPOP, FOXA1 and IDH1) were selected for comparison (the “Other” group). As we noticed substantial heterogeneity within the “Other” group, we conducted k-means clustering based on single sample gene set enrichment scores (GSVA(84)) of hallmark pathways (85), and divided it into two groups. One group seemed to have fewer CNV events than the other and was used as a pure “wild-type” reference group in differential analysis. Differential analysis between molecular subtypes of interest (example: FOXA1 Class 1 and PI3K altered) and the wild-type group was performed with Limma after filtering out lowly expressed genes and log-transformation of the TPM matrix. The human FOXA1 Class 1 tumor signature was defined as top 300 up-regulated genes (ranked by p-value) compared to the pure wild-type tumors. The mouse FOXA1 Class 1 tumor signature was derived from differential analysis of pseudo-bulk profiles of the tumor cell cluster of FOXA1 Class1 versus luminal cells of the wild-type (single cell RNA-seq data); genes with higher expression in basal cells (by 2-fold) than in luminal cells were excluded; top 300 genes ranked by p-value were then selected as the respective human and mouse signatures.

For SU2C RNA-seq analysis, transcriptomic data from an extended SU2C cohort (n=757) were downloaded in the form of RPKM data. FOXA1 class 1 cases were identified by an in-house pipeline (83)(86). PI3K pathway alteration was defined by pathogenic mutations in AKT1, PIK3CA, PIK3CB, or PTEN losses. The wild-type control group was defined as samples lacking alterations in ETS, SPOP, FOXA1, IDH1, and PI3K pathway genes. Differential expression analysis was performed using limma-trend with empirical Bayes moderation. The top 200 significantly ranked genes underwent Gene Ontology enrichment analysis via clusterProfiler v4.10.1 using default parameters (87). All statistical analyses were conducted in R v4.3.3. Data visualization employed ggplot2 v3.5.1 and ggpubr v0.6.0, with boxplots displaying median ± interquartile range.

Similar transcriptomic analysis from Asian primary PCa was performed in the CPGEA cohort. FOXA1 Class 1 cases were identified using a published pipeline (8). PI3K-altered cases include cases with PTEN mono and biallaelic losses, AKT mutations or PIK3CA amplifications and hotspot mutations. Cases that are not grouped into the major molecular subtypes (SPOP, MAP3K7, BRAF) were selected for comparison as the “Other” group. Next, DEG methodology was used to compare transcriptomes between FOXA1 Class 1 and PTEN/AKT-altered primary PCa. Briefly, we first identified DEGs in the PI3K-mutant vs common “Other” tumor sub-group (i.e., cases without FOXA1 Class 1 or PI3K alterations omitting MAP3K7 deletions, BRAF mutations and SPOP mutant cases). DEGs were also identified for FOXA1-Class 1 mutant vs the same “Other” tumor subtype and statistical significance was assessed using the prop.test. Next, to generate the transcriptomic concordance plot shown below, we plotted the fold-change of all DEGs (using the following threshold −1.5>log2FC>1.5) to assess transcriptomic concordance between FOXA1 Class1 and PI3K-altered cases. For GSEA analysis, we ranked genes based on their log₂FC values from the Class 1 (n=56) vs. Wildtype (n=62, omitting PI3K-altered cases, MAP3K7 deletions, BRAF mutations and SPOP mutant cases) comparison and performed GSEA analysis for hallmark AR signaling pathway.

TCGA PRAD protein analysis: Normalized RPPA value was downloaded from cBioportal (https://www.cbioportal.org/)(88). Boxplots of selected proteins in different molecular subtypes were generated with ggplot (89) and ggpubr (90); the non-parametric Wilcoxon signed-rank test was used for two-group comparison.

Asian primary PCa protein analysis: The phosphoproteomic data were obtained from the Figshare repository (DOI: 10.6084/m9.figshare.25706982)(31)(91). FOXA1 class 1 cases were identified by an in-house established pipeline (86). Cases with canonical alterations in ETS fusion genes, SPOP mutations, FOXA1 mutations outside Class 1, and IDH1 mutations were excluded. Remaining samples constituted the “Other” comparison group. Differential phosphorylation between FOXA1 Class 1 and Other groups was assessed using two-tailed Student’s t-tests. Boxplots of selected proteins in different molecular subtypes were generated with ggplot (89) and ggpubr (90)

Signature activity scores of KLF5 and Club signatures were calculated using the Single Sample Gene Set Enrichment Analysis (ssGSEA) method using the GSVA package in R. FOXA1 Class 2 clinical cases were stratified into Klf5 high and low groups based on a bimodal distribution of the CRPC_KLF5 signature. Here, we selected all samples with the Class 2 mutation and ssgsea activity of above 0.45 as KLF-high, and any Class 2 samples with ssgsea lower than 0.45 as KLF5-low.

### Tissue and organoid histology

Prostate tissue was fixed in 10% neutral buffered formalin overnight, dehydrated with ethanol, paraffin embedded, and serial sectioned at 4 μm thickness. Organoids were seeded at a density of 20,000 cells per 40 μL Matrigel dome and incubated in complete mouse prostate culture media for 5 to 7 days. Subsequently, organoid samples were harvested, embedded in Histogel, and fixed in 4% paraformaldehyde (PFA) for 1 hour, followed by dehydration with ethanol, paraffin embedding, and serial sectioning at five μm thickness.

### Histological analysis

Two genitourinary pathologists examined H&E-stains from formalin-fixed paraffin-embedded (FFPE) tissue sections in a blinded manner. Histological grading was conducted following a pre-approved schema at the beginning of the study (49). Each biopsy was assessed based on five broad histological categories through microscopic evaluation. These categories were as follows: Category 0 - indicating unremarkable prostate histology; Category 1 - indicating hyperplasia; Category 2 - indicating low and high-grade prostatic intraepithelial neoplasia (PIN); Category 3 - indicating florid high-grade PIN/atypical intraductal proliferation (AIP)/intraductal carcinoma-like; and category 4 - indicating invasive adenocarcinoma prostate (fig. S1H). Each prostate sample was scored by assigning a percentage prevalence to the five subcategories. The total score was then assigned to each biopsy and calculated as the sum of the percentage of cells in different subcategories (PC) with a score of 0 to 4. The total score (out of a possible 400) was calculated using the formula: TotalScore=(PCCategory0*0+PCCategory1*1+PCCategory2*2+PCCategory3*3+PCCategory4*4).

### Immunohistochemistry

Immunohistochemistry was performed manually or using the Ventana automated staining system (Roche Tissue Diagnostics). Antibodies used for immunohistochemistry are as follows: Ki67 (BD Pharmingen^™^: 550609); V5 tag (Thermo Fisher Scientific: R960–25); AR (Millipore: 06–680); FOXA1 (Thermo Fisher Scientific: PA5–27157); TROP2 (Bio-Techne R&D Systems: BAF1122); SMA (Roche-Ventana Medical Systems, 760–2833); p53 (Leica: P53-CM5P-L).

Manual: Stained slides were deparaffinized and incubated with a Citrate-based Antigen Unmasking Solution (Vector Laboratories, H-3300–250). 3% hydrogen peroxide (Sigma) was used to inactivate endogenous peroxidases. Primary antibodies were diluted in either normal horse serum (Vector Laboratories, S-2012–50) or 10% normal goat serum (Vector Laboratories, S-1000–20) and incubated overnight. Antibodies were detected with DAB Peroxidase Substrate kit (Vector Labs). The slides were counterstained with hematoxylin and cover-slipped with EcoMount (BioCare Medical).

Ventana: Ventana staining was carried out using a previously described protocol (10). Briefly, singleplex IHC was carried out on the Ventana ULTRA automated slide staining system (Roche-Ventana Medical Systems) using the OmniView Universal Diaminobenzidine Detection Kit (catalog number 760–500, Roche-Ventana) and Hematoxylin II (catalog number 790–2208, Roche-Ventana) for counterstain. Staining was evaluated under 10x and 20x magnification using a bright-field microscope.

### Multiplex Immunofluorescence (IF)

Multiplex IF was performed following a protocol described previously (10). The consecutive IF detection system was developed using the Discovery FITC Kit (catalog number 760–232, Roche-Ventana), Discovery Red 610 (catalog number 760–245), and the Discovery Cy5 Kit (catalog number 760–238, Roche-Ventana) with DAPI (Prolong Gold anti-fade, catalog number P36931, Invitrogen/Fisher-Scientific). Imaging of multiplex staining was performed using the EVOS 7000 system. Antibodies used for immunofluorescence were as follows: CK8 (Abcam: ab53280); AR (Ventana: 760–4605); V5 tag (Thermo Fisher Scientific: R960–25); TROP2 (Bio-Techne R&D Systems: BAF1122); NSD2 (Abcam: ab75359); NKX3.1 (Athena Enzyme Systems/Athena Environmental Sciences 0317). Fluorescent imaging were performed at 10x and 20x magnification.

## Supplementary Material

TableS3

TableS2

TableS1

4

## Data Availability

All data are available in the manuscript or the supplementary information. Raw next-generation sequencing data, including ChIP-seq, RNA-seq, and single-cell RNA/ATAC-seq, generated in this study are deposited in the Gene Expression Omnibus (GEO) repository (accession number: GSE282406) at National Center for Biotechnology Information. The code can be found at Zenodo (92) and (93).
