# Supplementary material for: Divergent FOXA1 mutations drive prostate tumorigenesis and therapy-resistant cellular plasticity
Source: Science. Author manuscript; Available in PMC 2025 Aug 6. (PMC12326538; doi:10.1126/science.adv2367)
Supplement: 4 [file NIHMS2097562-supplement-4.pdf]

Science
AAAS

Supplementary Materials for

Divergent FOXA1 mutations drive prostate tumorigenesis and therapy-resistant cellular plasticity

Sanjana Eyunni, Rahul Mannan, Yuping Zhang, Eleanor Young, Qiuyang Zhang, Jie Luo, Matthew Pang, Somnath Mahapatra, Jean Ching-Yi Tien, James M. George, Mustapha Jaber, Hamzah Hakkani, Sandra E. Carson, Abigail J. Todd, Noshad Hosseini, Mahnoor Gondal, Ryan J. Rebernick, Xuhong Cao, Fengyun Su, Rui Wang, Rohit Mehra, Jing Li, Marcin Cieslik, Arul M. Chinnaiyan, and Abhijit Parolia

Corresponding author: Arul M. Chinnaiyan, arul@med.umich.edu; Abhijit Parolia, aparolia@med.umich.edu

The PDF file includes:

Figs. S1 to S11
Captions for Tables S1 to S3

Other Supplementary Materials for this manuscript include the following:

Tables S1 to S3
MDAR Reproducibility Checklist

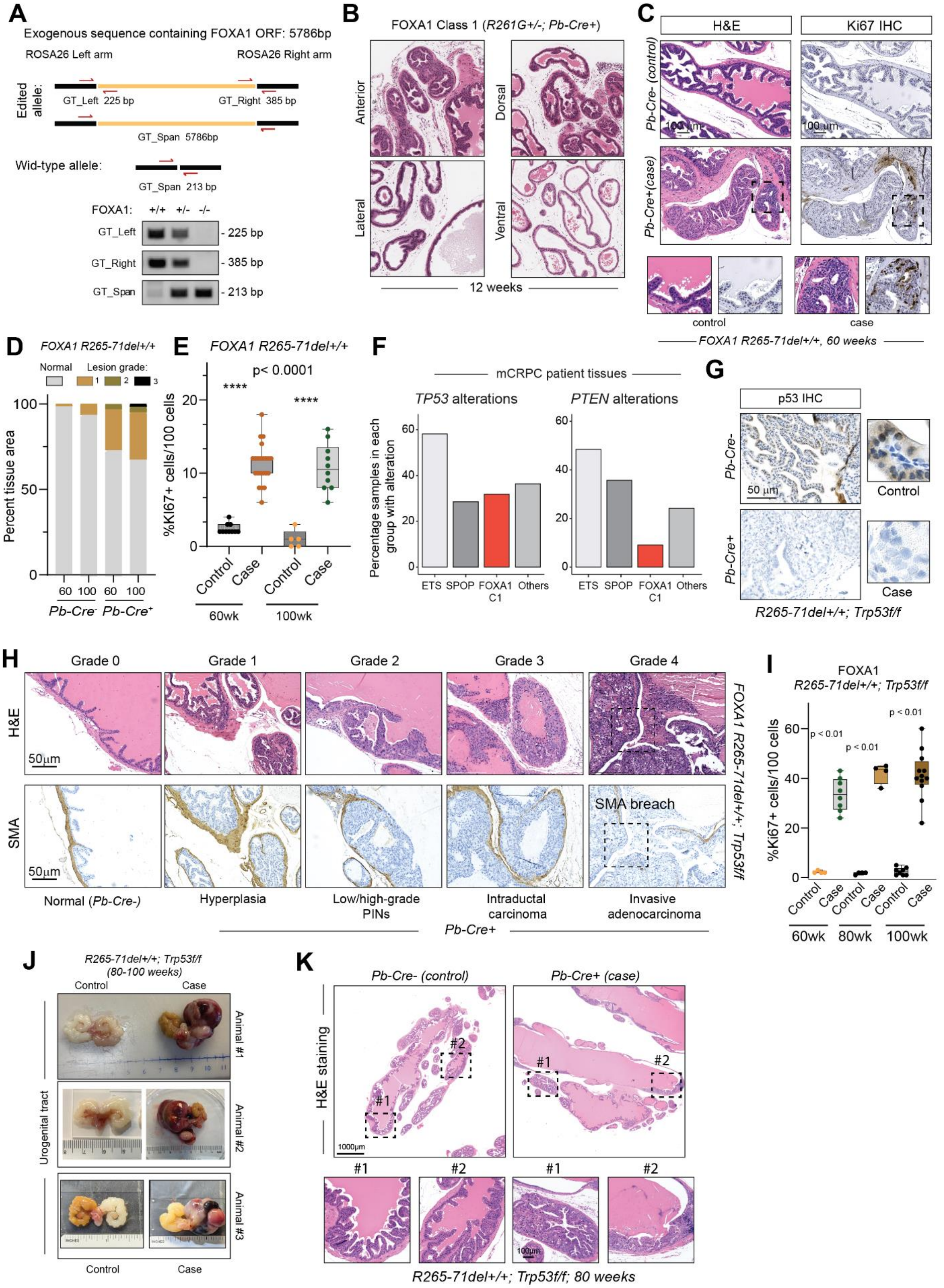
A
Exogenous sequence containing FOXA1 ORF: 5786bp
ROSA26 Left arm
ROSA26 Right arm
Edited allele:
GT_Left 225 bp
GT_Right 385 bp
GT_Span 5786bp
Wid-type allele:
GT_Span 213 bp
FOXA1: +/+ +/- -/-
GT_Left - 225 bp
GT_Right - 385 bp
GT_Span - 213 bp
B
FOXA1 Class 1 (R261G+/-; Pb-Cre+)
Anterior
Dorsal
Lateral
Ventral
12 weeks
C
H&E
Ki67 IHC
Pb-Cre- (control)
Pb-Cre+(case)
100 μm
control
case
FOXA1 R265-71del+/+, 60 weeks
D
FOXA1 R265-71del+/+
Normal
Lesion grade: 1 2 3
Percent tissue area
60 100 60 100
Pb-Cre⁻ Pb-Cre⁺
E
FOXA1 R265-71del+/+
p< 0.0001
****
%Ki67+ cells/100 cells
Control Case Control Case
60wk
100wk
F
mCRPC patient tissues
TP53 alterations
PTEN alterations
Percentage samples in each group with alteration
ETS SPOP FOXA1 C1 Others
G
p53 IHC
Pb-Cre-
Pb-Cre+
50 μm
Control
Case
R265-71del+/+; Trp53f/f
H
Grade 0
Grade 1
Grade 2
Grade 3
Grade 4
H&E
SMA
50μm
SMA breach
FOXA1 R265-71del+/+; Trp53f/f
Normal (Pb-Cre-)
Hyperplasia
Low/high-grade PINs
Intraductal carcinoma
Invasive adenocarcinoma
Pb-Cre+
I
FOXA1
R265-71del+/+; Trp53f/f
%Ki67+ cells/100 cells
p < 0.01
Control Case Control Case Control Case
60wk 80wk 100wk
J
R265-71del+/+; Trp53f/f
(80-100 weeks)
Control
Case
Urogenital tract
Animal #1
Animal #2
Animal #3
K
Pb-Cre- (control)
Pb-Cre+ (case)
H&E staining
#1
#2
1000μm
100μm
R265-71del+/+; Trp53f/f; 80 weeks

Fig. S1. Generation and characterization of FOXA1 Class 1 mutant transgenic mice. A) Top: Schematic of the FOXA1 knock-in construct at the ROSA26 locus with genotyping primers highlighted in red. Bottom: Representative genotyping agarose gel distinguishing transgenic mice that are wild-type (-/-), heterozygous (+/-), or homozygous (+/+). GTL and GTR bands indicate the presence of the transgene, while the absence of a span band indicates a homozygous mutant animal. B) Representative H&E-stained cross-sections of *FOXA1 R261G+/-* Cre+ case prostate lobes from 12-week-old mice. Scale =100µm. C) Representative H&E and Ki67 stained cross-sections of *FOXA1 R265-71del+/+* control and case prostate tissues (anterior lobe) from 60-week-old mice. Scale =100µm. D) Histopathological grading of tissues from panel C. E) Percentage of Ki67-positive cells from 60 and 100-week-old tissues from *FOXA1 R265-71del+/+* control-case prostate tissues (two-sided t-test). Box plot: center line, median; box, interquartile range (Q1–Q3); whiskers, minimum to maximum values; all individual data points shown. F) Co-recurrence of *TP53* or *PTEN* alterations in CRPC tumors harboring mutually exclusive ETS fusions, SPOP mutations, or FOXA1 Class 1 mutations (n=371). G) Representative p53 IHC stained cross-sections of *FOXA1 R265-71del+/+;Trp53f/f* control and case animals. Scale =50µm. H) Representative H&E and alpha-SMA stained cross sections in the *FOXA1 R265-71del+/+;Trp53f/f* tissues representing the distinct grades used in this study. SMA staining is retained and highlighted as continuous brown expression at the parabasal regions in Grade 1:Hyperplasia, Grade 2: Low/high-grade prostate intraepithelial neoplasia (PINs), Grade 3: Florid high-grade PIN, Atypical intraductal proliferation and Intraductal carcinoma, but absent in Grade 4: Invasive adenocarcinoma. I) Percentage of Ki67-positive cells from 60, 80, and 100 week-old tissues from *FOXA1 R265-71del+/+;Trp53f/f* control-case prostate tissues (two-sided t-test). Box plot: center line, median; box, interquartile range (Q1–Q3); whiskers, minimum to maximum values; all individual data points shown. J) Representative images of the urogenital tract (prostate gland and seminal vesicles) in three distinct FOXA1 *R265-71del+/+;Trp53f/f* animals. K) Representative H&E-stained cross-sections of *FOXA1 R265-71del+/+;Trp53f/f* control-case prostate tissues (anterior lobe) from 80 week-old mice. Scale =1000um. Zoomed insets show normal and tumor lesions. Scale =100um.

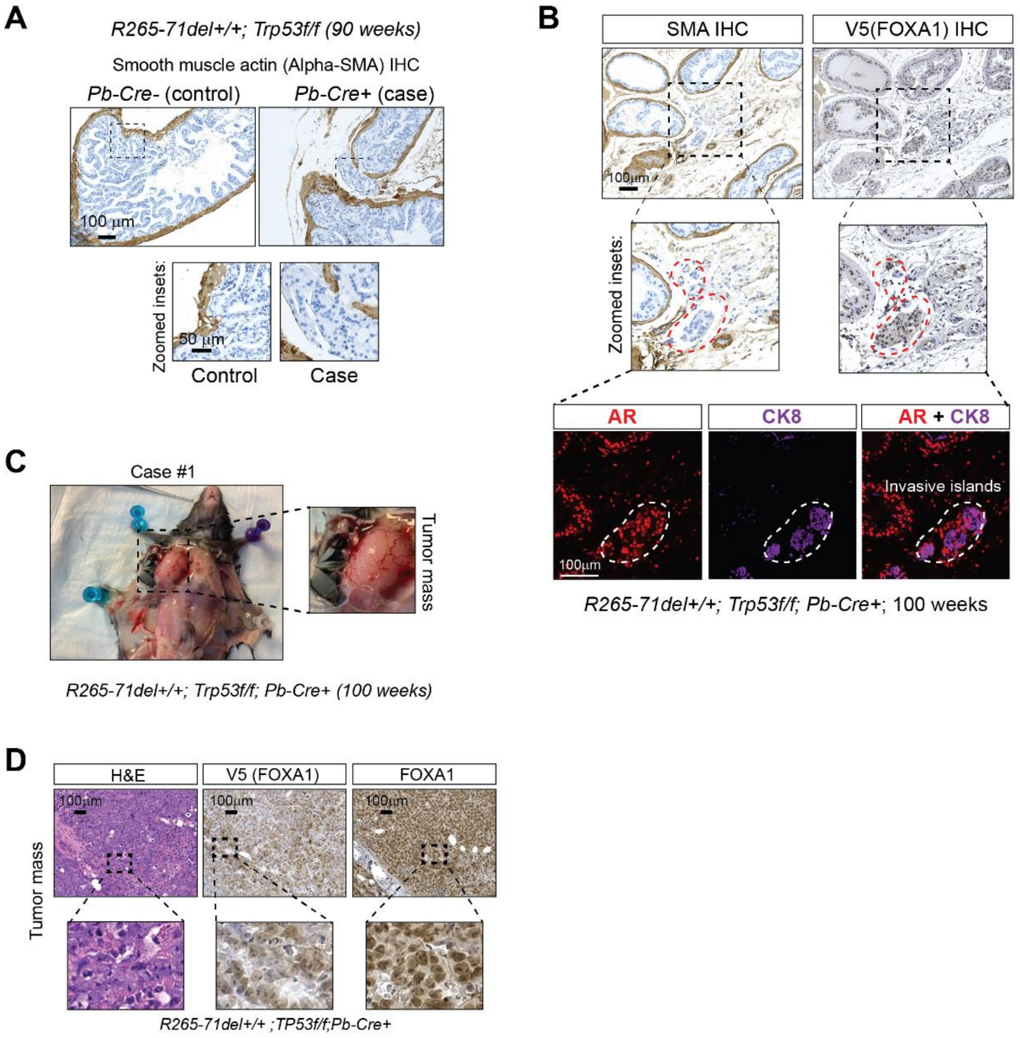
A
R265-71del+/+; Trp53f/f (90 weeks)
Smooth muscle actin (Alpha-SMA) IHC
Pb-Cre- (control)
Pb-Cre+ (case)
100 µm
Zoomed insets:
50 µm
Control
Case
B
SMA IHC
V5(FOXA1) IHC
100µm
Zoomed insets:
AR
CK8
AR + CK8
Invasive islands
100µm
R265-71del+/+; Trp53f/f; Pb-Cre+; 100 weeks
C
Case #1
Tumor mass
R265-71del+/+; Trp53f/f; Pb-Cre+ (100 weeks)
D
H&E
V5 (FOXA1)
FOXA1
100µm
100µm
100µm
Tumor mass
R265-71del+/+ ;TP53f/f;Pb-Cre+

Fig. S2. High-grade invasive carcinoma in *FOXA1 R265-71del+/+;Trp53f/f* animals. A) Representative alpha-SMA IHC in *FOXA1 R265-71del+/+;Trp53f/f* 90-week-old control and case tissues. Scale =100µm. Zoomed insets of selected regions (highlighted in a black, dotted box) are shown below. B) Representative alpha-SMA and V5 IHC and multiplex- IF of CK8 and AR in *FOXA1 R265-71del+/+;Trp53f/f* 100-week-old case tissues. Zoomed insets of delaminated/invasive regions (highlighted in a black, dotted box and red, dotted circles) are shown below. Scale =100µm. C) Image of a tumor mass in a 100-week-old *FOXA1 R265-71del+/+;Trp53f/f* case animal. D) Representative H&E, V5, and FOXA1 stained cross sections from the tumor cavity mass shown in panel C. Scale =100µm.

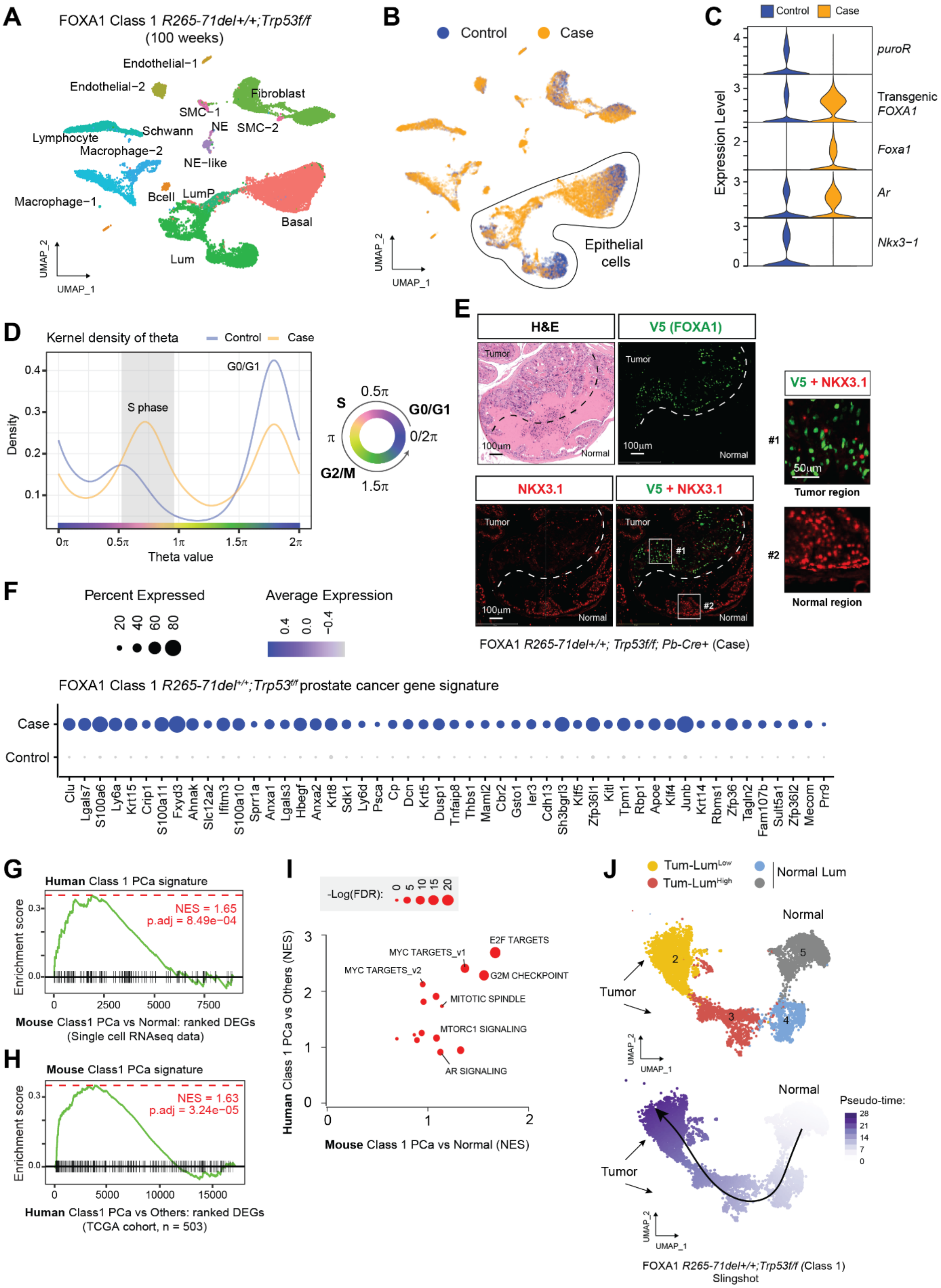
A
FOXA1 Class 1 R265-71del+/+;Trp53f/f
(100 weeks)
Endothelial−1
Endothelial−2
Fibroblast
SMC−1
Schwann
NE
SMC−2
Lymphocyte
Macrophage−2
NE−like
Macrophage−1
Bcell
LumP
Basal
Lum
UMAP_2
UMAP_1
B
Control
Case
Epithelial cells
UMAP_2
UMAP_1
C
Control
Case
Expression Level
puroR
Transgenic FOXA1
Foxa1
Ar
Nkx3−1
D
Kernel density of theta
Control
Case
Density
S phase
G0/G1
Theta value
0.5π
S
G0/G1
π
0/2π
G2/M
1.5π
E
H&E
V5 (FOXA1)
NKX3.1
V5 + NKX3.1
Tumor
Normal
100μm
#1
#2
50μm
Tumor region
Normal region
FOXA1 R265-71del+/+; Trp53f/f; Pb-Cre+ (Case)
F
Percent Expressed
Average Expression
FOXA1 Class 1 R265-71del+/+;Trp53f/f prostate cancer gene signature
Case
Control
Clu
Lgals7
S100a6
Ly6a
Krt15
Crip1
S100a11
Fxyd3
Ahnak
Slc12a2
Ifitm3
S100a10
Sprr1a
Anxa1
Lgals3
Hbegf
Anxa2
Krt8
Sdk1
Ly6d
Psca
Cp
Dcn
Krt5
Dusp1
Tnfaip8
Thbs1
Maml2
Cbr2
Gsto1
Ier3
Cdh13
Sh3bgrl3
Klf5
Zfp36l1
Kitl
Tpm1
Rbp1
Apoe
Klf4
Junb
Krt14
Rbms1
Zfp36
Tagln2
Fam107b
Sult5a1
Zfp36l2
Mecom
Prr9
G
Human Class 1 PCa signature
Enrichment score
NES = 1.65
p.adj = 8.49e−04
Mouse Class1 PCa vs Normal: ranked DEGs
(Single cell RNAseq data)
H
Mouse Class1 PCa signature
Enrichment score
NES = 1.63
p.adj = 3.24e−05
Human Class1 PCa vs Others: ranked DEGs
(TCGA cohort, n = 503)
I
-Log(FDR):
Human Class 1 PCa vs Others (NES)
E2F TARGETS
MYC TARGETS_v1
MYC TARGETS_v2
G2M CHECKPOINT
MITOTIC SPINDLE
MTORC1 SIGNALING
AR SIGNALING
Mouse Class 1 PCa vs Normal (NES)
J
Tum-Lum^Low
Tum-Lum^High
Normal Lum
Normal
Tumor
UMAP_2
UMAP_1
Pseudo-time:
FOXA1 R265-71del+/+;Trp53f/f (Class 1)
Slingshot

Fig. S3. Single-cell transcriptomic characterization of *FOXA1 R265-71del+/+;Trp53f/f* tumor tissues. A) UMAP plots of the distinct cell types present in the 100-week-old *FOXA1 R265-71del+/+;Trp53f/f* case and control animals (10x single-cell RNA-sequencing). B) UMAP plots of the distinct cell types from panel (A) with case or control tissue-derived cells colored across clusters. Epithelial cells used for further analyses are marked with a solid black line. C) Violin plots showing expression of select genes in the epithelial cells from control or case *FOXA1 R265-71del+/+;Trp53f/f* animals. D) Cell cycle plot (using the Tricycle R package) shows distribution of case or control cells across the distinct cell cycle phases as denoted by the theta values. E) Representative H&E and multiplex-IF of V5 and NKX3.1 in *FOXA1 R265-71del+/+;Trp53f/f* case tissues. Normal and tumor regions are annotated along with zoomed insets. F) Dot plot of the prostate cancer signature genes (defined in this study, see methods) between *FOXA1 R265-71del+/+;Trp53f/f* case and control cells. G) Gene set enrichment analysis (GSEA) plots for human Class 1 PCa activated signature from the rank-ordered genes from mouse Class 1 mutant tumor tissues. DEGS, differentially expressed genes (GSEA enrichment test). H) Gene set enrichment analysis (GSEA) plots for mouse Class 1 PCa activated signature from the rank-ordered genes of human Class 1 mutant tumor tissues. DEGS, differentially expressed genes (GSEA enrichment test). I) Hallmark gene set net enrichment score (NES) between mouse and human Class 1 ranked gene lists. Each point denotes a hallmark gene set, and the size of the points corresponds to the false discovery rate (FDR). J) UMAP plot of distinct clusters in the 100-week-old *FOXA1 R265-71del+/+;Trp53f/f* single-cell RNA-seq data (Top, basal cells removed) and pseudotime trajectory analyses (Bottom, Slingshot, see methods). The solid black arrow denotes the trajectory from normal (cluster 4,5) to tumor populations (cluster 2,3).

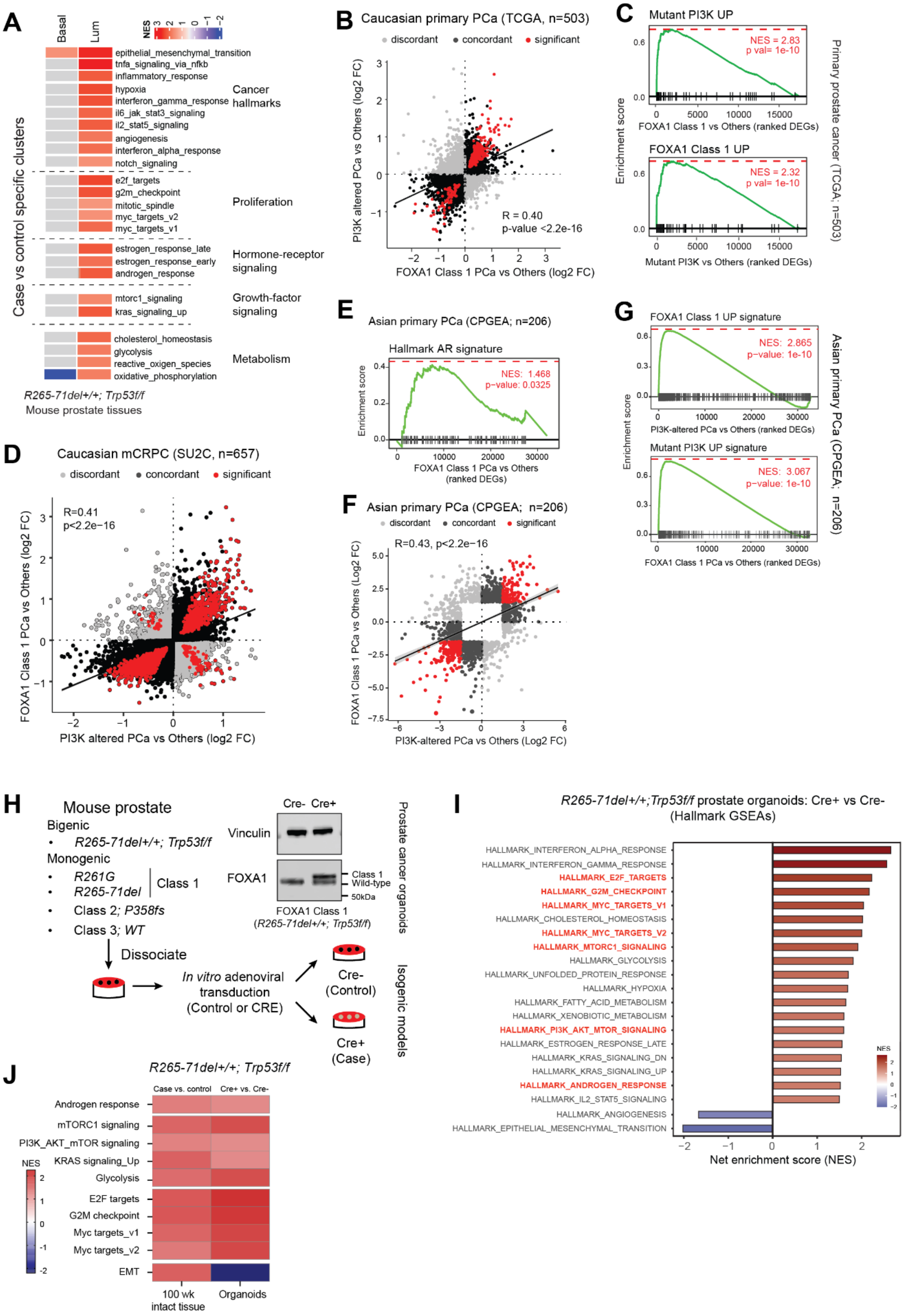
A
Basal
Lum
NES
3 2 1 0 −1 −2
epithelial_mesenchymal_transition
tnfa_signaling_via_nfkb
inflammatory_response
hypoxia
interferon_gamma_response
il6_jak_stat3_signaling
il2_stat5_signaling
angiogenesis
interferon_alpha_response
notch_signaling
Cancer hallmarks
e2f_targets
g2m_checkpoint
mitotic_spindle
myc_targets_v2
myc_targets_v1
Proliferation
estrogen_response_late
estrogen_response_early
androgen_response
Hormone-receptor signaling
mtorc1_signaling
kras_signaling_up
Growth-factor signaling
cholesterol_homeostasis
glycolysis
reactive_oxigen_species
oxidative_phosphorylation
Metabolism
Case vs control specific clusters
R265-71del+/+; Trp53f/f
Mouse prostate tissues
B
Caucasian primary PCa (TCGA, n=503)
discordant
concordant
significant
R = 0.40
p-value <2.2e-16
PI3K altered PCa vs Others (log2 FC)
FOXA1 Class 1 PCa vs Others (log2 FC)
C
Mutant PI3K UP
NES = 2.83
p val= 1e-10
FOXA1 Class 1 vs Others (ranked DEGs)
FOXA1 Class 1 UP
NES = 2.32
p val= 1e-10
Mutant PI3K vs Others (ranked DEGs)
Enrichment score
Primary prostate cancer (TCGA; n=503)
D
Caucasian mCRPC (SU2C, n=657)
discordant
concordant
significant
R=0.41
p<2.2e-16
FOXA1 Class 1 PCa vs Others (log2 FC)
PI3K altered PCa vs Others (log2 FC)
E
Asian primary PCa (CPGEA; n=206)
Hallmark AR signature
NES: 1.468
p-value: 0.0325
Enrichment score
FOXA1 Class 1 PCa vs Others (ranked DEGs)
F
Asian primary PCa (CPGEA; n=206)
discordant
concordant
significant
R=0.43, p<2.2e-16
FOXA1 Class 1 PCa vs Others (Log2 FC)
PI3K-altered PCa vs Others (Log2 FC)
G
FOXA1 Class 1 UP signature
NES: 2.865
p-value: 1e-10
PI3K-altered PCa vs Others (ranked DEGs)
Mutant PI3K UP signature
NES: 3.067
p-value: 1e-10
FOXA1 Class 1 PCa vs Others (ranked DEGs)
Enrichment score
Asian primary PCa (CPGEA; n=206)
H
Mouse prostate
Bigenic
R265-71del+/+; Trp53f/f
Monogenic
R261G
R265-71del
Class 1
Class 2; P358fs
Class 3; WT
Dissociate
In vitro adenoviral transduction (Control or CRE)
Cre- (Control)
Cre+ (Case)
Cre- Cre+
Vinculin
FOXA1
Class 1
Wild-type
50kDa
FOXA1 Class 1 (R265-71del+/+; Trp53f/f)
Prostate cancer organoids
Isogenic models
I
R265-71del+/+;Trp53f/f prostate organoids: Cre+ vs Cre- (Hallmark GSEAs)
HALLMARK_INTERFERON_ALPHA_RESPONSE
HALLMARK_INTERFERON_GAMMA_RESPONSE
HALLMARK_E2F_TARGETS
HALLMARK_G2M_CHECKPOINT
HALLMARK_MYC_TARGETS_V1
HALLMARK_CHOLESTEROL_HOMEOSTASIS
HALLMARK_MYC_TARGETS_V2
HALLMARK_MTORC1_SIGNALING
HALLMARK_GLYCOLYSIS
HALLMARK_UNFOLDED_PROTEIN_RESPONSE
HALLMARK_HYPOXIA
HALLMARK_FATTY_ACID_METABOLISM
HALLMARK_XENOBIOTIC_METABOLISM
HALLMARK_PI3K_AKT_MTOR_SIGNALING
HALLMARK_ESTROGEN_RESPONSE_LATE
HALLMARK_KRAS_SIGNALING_DN
HALLMARK_KRAS_SIGNALING_UP
HALLMARK_ANDROGEN_RESPONSE
HALLMARK_IL2_STAT5_SIGNALING
HALLMARK_ANGIOGENESIS
HALLMARK_EPITHELIAL_MESENCHYMAL_TRANSITION
NES
Net enrichment score (NES)
J
R265-71del+/+; Trp53f/f
Case vs. control
Cre+ vs. Cre-
Androgen response
mTORC1 signaling
PI3K_AKT_mTOR signaling
KRAS signaling_Up
Glycolysis
E2F targets
G2M checkpoint
Myc targets_v1
Myc targets_v2
EMT
NES
100 wk intact tissue
Organoids

Fig. S4. FOXA1 Class 1 tumors are enriched for hyper-proliferative and oncogenic signatures. A) Heatmap of significantly enriched hallmark pathways from single-cell RNA-seq of *FOXA1 R265-71del+/+;Trp53f/f* tissues. Enriched pathways are clubbed by distinct cancer-relevant themes. B) Correlation plot of differentially expressed genes between the PI3K and FOXA1 class 1 mutant tumors from the TCGA primary PCa clinical cohort (n=503, pearson correlation test). C) Gene set enrichment analysis (GSEA) plots for PI3K and FOXA1 Class 1 activated genes from FOXA1 Class 1 or PI3K mutant human primary PCa samples (TCGA, n=503). DEGS, differentially expressed genes (GSEA enrichment test). D) Correlation plot of differentially expressed genes between the PI3K and FOXA1 class 1 mutant tumors from the SU2C CRPC PCa clinical cohort (n=657, pearson correlation test). E) GSEA plots of AR up-regulated genes using the fold change from Asian Class 1 mutant patient tumor tissues. DEGS, differentially expressed genes (n=206, GSEA enrichment test). F) Correlation plot of differentially expressed genes between the PI3K and FOXA1 class 1 mutant tumors from the CPGEA primary PCa clinical cohort (n=206, pearson correlation test). G) Gene set enrichment analysis (GSEA) plots for PI3K and FOXA1 Class 1 activated genes from FOXA1 Class 1 or PI3K mutant CPGEA patient tumor tissues. DEGS, differentially expressed genes (n=206, GSEA enrichment test). H) Left: Schematic of the *in vitro* generation of isogenic mouse prostate organoids. Control organoids denoted as Cre- do not express the FOXA1 mutants, while Cre+ organoids express the distinct mutations. Right: Immunoblots of noted proteins in bigenic (*R265-71del+/+;Trp53f/f)* cancer organoids. I) Heatmap of the net enrichment scores (NES) for noted hallmark pathways in the Cre+ vs Cre- *R265-71del+/+;Trp53f/f* prostate cancer organoids. J) Heatmap of NES scores showing concordance of noted cancer-relevant pathways between the FOXA1 *R265-71del+/+;Trp53f/f* tumor tissues (single-cell RNA seq) and tumor organoids.

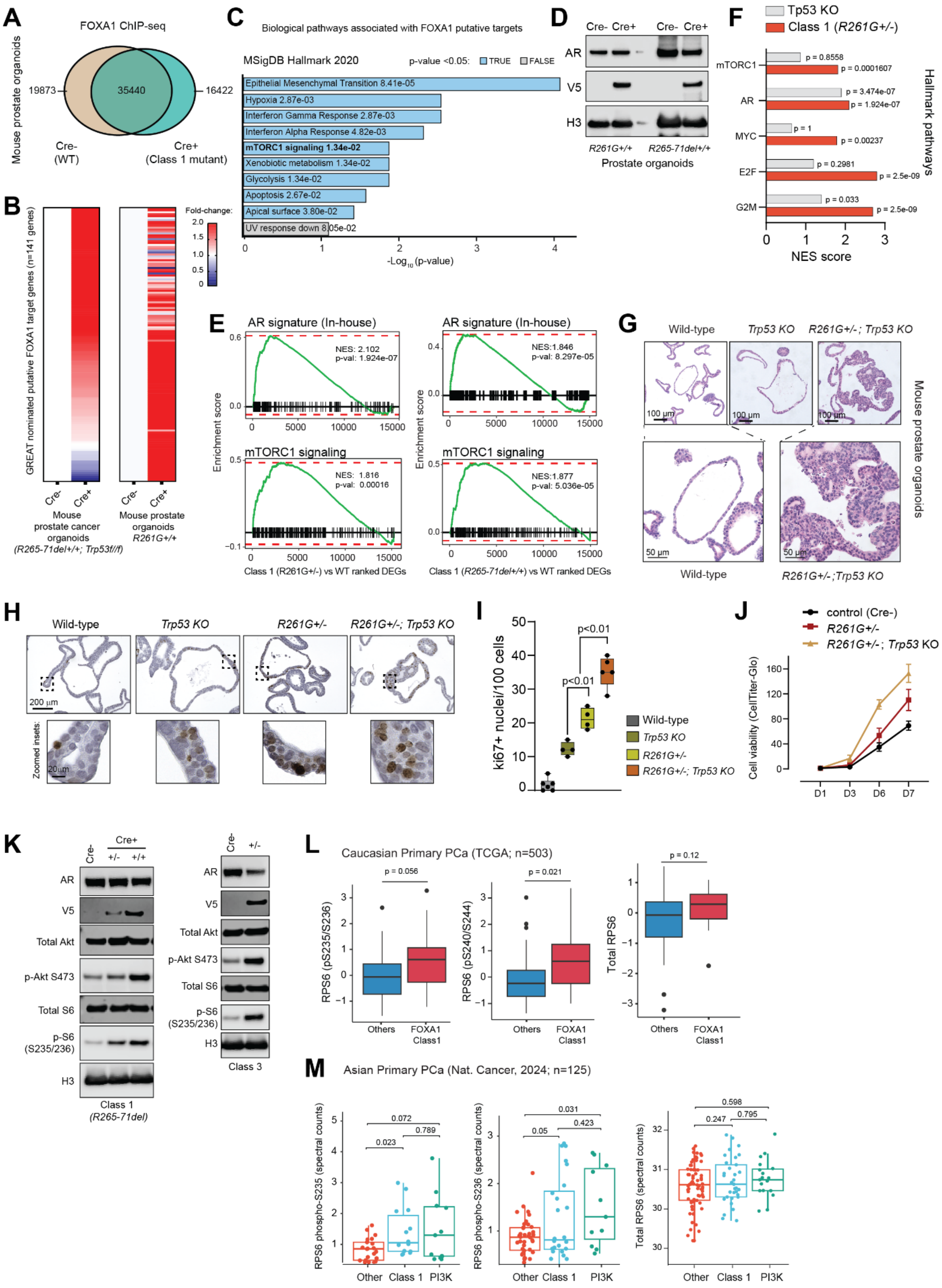
A
FOXA1 ChIP-seq
Mouse prostate organoids
19873
35440
16422
Cre- (WT)
Cre+ (Class 1 mutant)
B
GREAT nominated putative FOXA1 target genes (n=141 genes)
Fold-change:
Cre-
Cre+
Mouse prostate cancer organoids (R265-71del+/+; Trp53fl/fl)
Mouse prostate organoids R261G+/+
C
Biological pathways associated with FOXA1 putative targets
MSigDB Hallmark 2020
p-value <0.05: TRUE FALSE
Epithelial Mesenchymal Transition 8.41e-05
Hypoxia 2.87e-03
Interferon Gamma Response 2.87e-03
Interferon Alpha Response 4.82e-03
mTORC1 signaling 1.34e-02
Xenobiotic metabolism 1.34e-02
Glycolysis 1.34e-02
Apoptosis 2.67e-02
Apical surface 3.80e-02
UV response down 8.05e-02
-Log10 (p-value)
D
Cre- Cre+ Cre- Cre+
AR
V5
H3
R261G+/+
R265-71del+/+
Prostate organoids
F
Tp53 KO
Class 1 (R261G+/-)
mTORC1
AR
MYC
E2F
G2M
p = 0.8558
p = 0.0001607
p = 3.474e-07
p = 1.924e-07
p = 1
p = 0.00237
p = 0.2981
p = 2.5e-09
p = 0.033
p = 2.5e-09
NES score
Hallmark pathways
E
AR signature (In-house)
NES: 2.102
p-val: 1.924e-07
NES:1.846
p-val: 8.297e-05
mTORC1 signaling
NES: 1.816
p-val: 0.00016
NES:1.877
p-val: 5.036e-05
Enrichment score
Class 1 (R261G+/-) vs WT ranked DEGs
Class 1 (R265-71del+/+) vs WT ranked DEGs
G
Wild-type
Trp53 KO
R261G+/-; Trp53 KO
100 μm
50 μm
Wild-type
R261G+/-;Trp53 KO
Mouse prostate organoids
H
Wild-type
Trp53 KO
R261G+/-
R261G+/-; Trp53 KO
200 μm
Zoomed insets:
20 μm
I
ki67+ nuclei/100 cells
p<0.01
p<0.01
Wild-type
Trp53 KO
R261G+/-
R261G+/-; Trp53 KO
J
control (Cre-)
R261G+/-
R261G+/-; Trp53 KO
Cell viability (CellTiter-Glo)
D1
D3
D6
D7
K
Cre-
Cre+
+/-
+/+
AR
V5
Total Akt
p-Akt S473
Total S6
p-S6 (S235/236)
H3
Class 1 (R265-71del)
Cre-
+/-
Class 3
L
Caucasian Primary PCa (TCGA; n=503)
p = 0.056
p = 0.021
p = 0.12
RPS6 (pS235/S236)
RPS6 (pS240/S244)
Total RPS6
Others
FOXA1 Class1
M
Asian Primary PCa (Nat. Cancer, 2024; n=125)
0.072
0.023
0.789
0.031
0.05
0.423
0.598
0.247
0.795
RPS6 phospho-S235 (spectral counts)
RPS6 phospho-S236 (spectral counts)
Total RPS6 (spectral counts)
Other
Class 1
PI3K

Fig. S5. FOXA1 Class 1 mutants reprogram AR and activate mTORC1 signaling. A) Overlap of the wild-type and Class 1 mutant FOXA1 cistrome in the Class 1 Cre- and Cre+ mouse prostate organoids. B) Fold-change expression of GREAT-nominated genes in Class 1-mutant expressing normal mouse prostate or PCa organoids (RNA-seq, n = 2 biological replicates). C) Enrichr analyses of putative FOXA1 gene targets using biology pathways in the MSigDB database (Fisher's exact test). D) Immunoblot of noted proteins in the monogenic Class 1 prostate organoids (*R265-71del, R261G*). E) GSEA plots of AR and mTORC1 up-regulated genes using the fold change from *FOXA1 R261G* (left) and *R265-71del* (right) Cre+ vs Cre- tumor organoids. DEGS, differentially expressed genes. (n=2 biological replicates, GSEA enrichment test). F) Barplots of the NES scores for the labeled hallmark pathways from RNA-seq data of *Trp53 KO* or FOXA1 Class 1 *R261G+/-* mouse prostate organoids (GSEA enrichment test). G) Representative H&E images of the wild-type, *Trp53* knock-out (KO), and *FOXA1 R261G+/-;Trp53* KO mouse prostate organoids. Scale=100µm; inset scale =50µm. H) Representative Ki67 IHC images of the distinct *R261G* isogenic mouse prostate organoids. I) Semi-quantification using percentage positivity of nuclear Ki67 expression in the four sub-cohorts shown in panel H (unpaired t-test. Box plot: center line, median; box, interquartile range (Q1–Q3); whiskers, minimum to maximum values; all individual data points shown). J) Cell growth (Cell Titer Glow) plots of the control, *R261G+/-* or *R261G+/-;Trp53 KO* organoid lines in complete media (n= 4 biological replicates). K) Immunoblots of labeled proteins in Cre- and Cre+ Class 1 (*R265-71del*, left) and Class 3 (wild-type overexpression, right) mouse prostate organoids. L) Reverse phase protein array-based expression of phosphorylated RPS6 (Serine 235 and 236) and total RPS6 in FOXA1 Class 1 mutant tumors from TCGA primary PCa samples (n=503, wilcoxon signed-rank test). M) Protein expression levels of phosphorylated RPS6 (Serine 235 and Serine 236) and total RPS6 in FOXA1 Class 1 and PI3K mutant tumors from Asian primary PCa samples (n=125, student t-test).

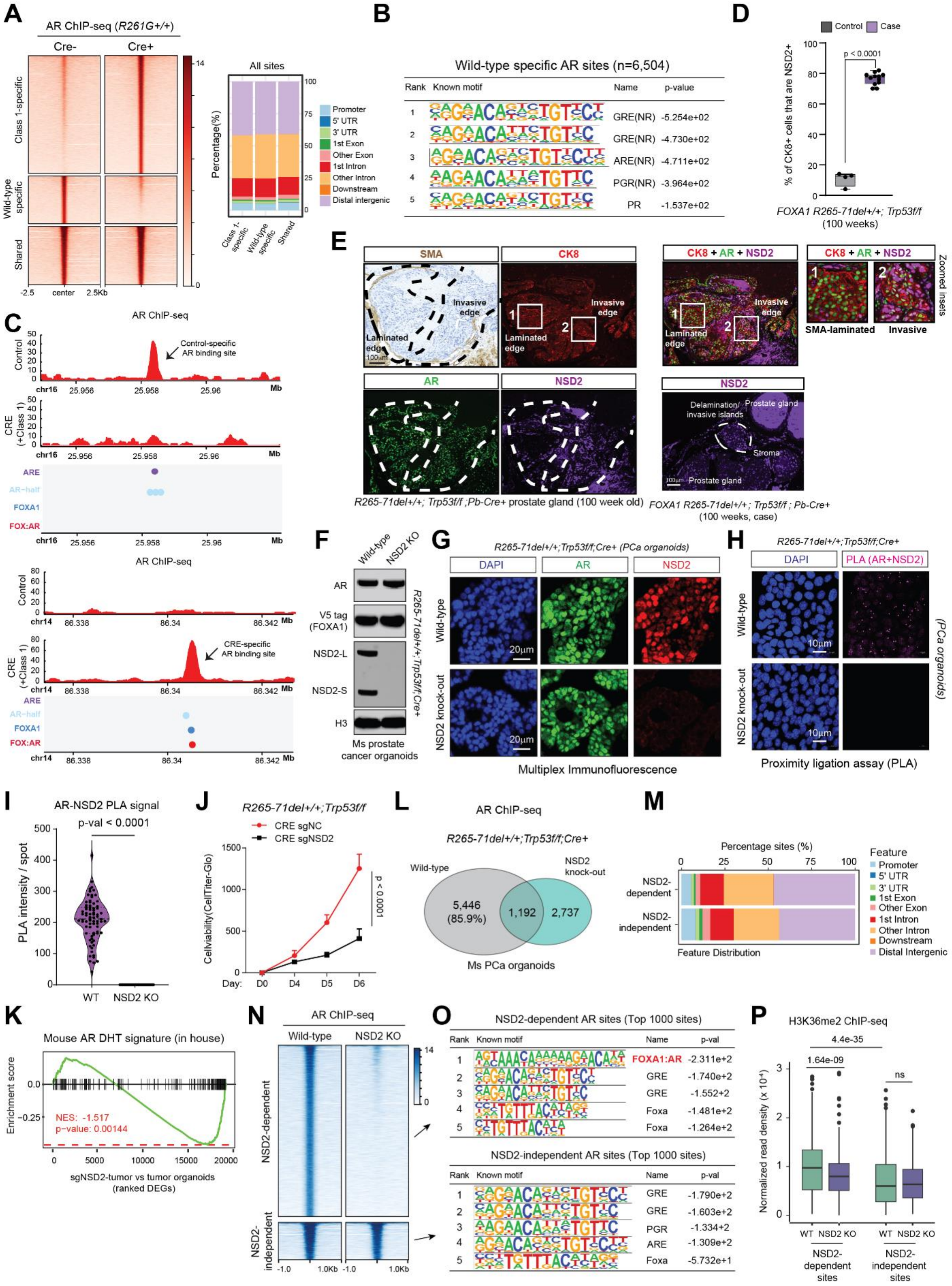
A
AR ChIP-seq (R261G+/+)
Cre-
Cre+
Class 1-specific
Wild-type specific
Shared
-2.5
center
2.5Kb
All sites
Percentage(%)
Promoter
5' UTR
3' UTR
1st Exon
Other Exon
1st Intron
Other Intron
Downstream
Distal intergenic
B
Wild-type specific AR sites (n=6,504)
Rank
Known motif
Name
p-value
GRE(NR) -5.254e+02
GRE(NR) -4.730e+02
ARE(NR) -4.711e+02
PGR(NR) -3.964e+02
PR -1.537e+02
D
Control
Case
% of CK8+ cells that are NSD2+
p < 0.0001
FOXA1 R265-71del+/+; Trp53f/f
(100 weeks)
C
AR ChIP-seq
Control
Control-specific AR binding site
CRE (+Class 1)
ARE
AR-half
FOXA1
FOX:AR
CRE-specific AR binding site
E
SMA
CK8
Invasive edge
Laminated edge
CK8 + AR + NSD2
Zoomed insets
SMA-laminated
Invasive
AR
NSD2
Delamination/ invasive islands
Prostate gland
Stroma
R265-71del+/+; Trp53f/f ;Pb-Cre+ prostate gland (100 week old)
FOXA1 R265-71del+/+; Trp53f/f ; Pb-Cre+
(100 weeks, case)
F
Wild-type
NSD2 KO
AR
V5 tag (FOXA1)
NSD2-L
NSD2-S
H3
R265-71del+/+;Trp53f/f;Cre+
Ms prostate cancer organoids
G
R265-71del+/+;Trp53f/f;Cre+ (PCa organoids)
DAPI
AR
NSD2
Wild-type
NSD2 knock-out
20μm
Multiplex Immunofluorescence
H
R265-71del+/+;Trp53f/f;Cre+
DAPI
PLA (AR+NSD2)
10μm
(PCa organoids)
Proximity ligation assay (PLA)
I
AR-NSD2 PLA signal
p-val < 0.0001
PLA intensity / spot
WT
NSD2 KO
J
R265-71del+/+;Trp53f/f
CRE sgNC
CRE sgNSD2
Cellviability(CellTiter-Glo)
p < 0.0001
Day: D0 D4 D5 D6
L
AR ChIP-seq
R265-71del+/+;Trp53f/f;Cre+
Wild-type
NSD2 knock-out
5,446 (85.9%)
1,192
2,737
Ms PCa organoids
M
Percentage sites (%)
NSD2-dependent
NSD2-independent
Feature Distribution
Feature
Promoter
5' UTR
3' UTR
1st Exon
Other Exon
1st Intron
Other Intron
Downstream
Distal Intergenic
K
Mouse AR DHT signature (in house)
Enrichment score
NES: -1.517
p-value: 0.00144
sgNSD2-tumor vs tumor organoids
(ranked DEGs)
N
AR ChIP-seq
Wild-type
NSD2 KO
NSD2-dependent
NSD2-independent
-1.0
1.0Kb
O
NSD2-dependent AR sites (Top 1000 sites)
Rank
Known motif
Name
p-val
FOXA1:AR -2.311e+2
GRE -1.740e+2
GRE -1.552+2
Foxa -1.481e+2
Foxa -1.264e+2
NSD2-independent AR sites (Top 1000 sites)
GRE -1.790e+2
GRE -1.603e+2
PGR -1.334+2
ARE -1.309e+2
Foxa -5.732e+1
P
H3K36me2 ChIP-seq
4.4e-35
1.64e-09
ns
Normalized read density (x 10⁻⁴)
WT
NSD2 KO
NSD2-dependent sites
NSD2-independent sites

Fig. S6. NSD2 reprograms AR at chimeric AR half-sites. A) Left: ChIP-seq read-density heatmaps of AR at the Class 1-specific, wild-type, or shared AR binding sites in the *FOXA1 R261G+/+* mouse prostate organoids. Right: Genomic location of the wild-type, Class 1-specific, and shared AR binding sites from the heatmap in the left panel. B) Top five HOMER known motifs (ranked by p-value) enriched within Cre- i.e., wild-type specific AR binding sites. (HOMER, hypergeometric test). C) ChIP-seq read-density tracks of AR binding within loci in chromosome 16 and 14 in Cre- and Cre+ FOXA1 Class 1 *R261G+/+* prostate organoids. HOMER motifs detected within AR peaks are shown below. D) Boxplot showing the percentage of CK8+ cells that are NSD2+ cells in 100-week-old *FOXA1 R265-71del+/+;Trp53f/f* control and case tissues as quantified through multiplex-IF sections (two-sided t-test). Box plot: center line, median; box, interquartile range (Q1–Q3); whiskers, minimum to maximum values; all individual data points shown. E) Representative multiplex immunofluorescence of noted proteins in *FOXA1 R265-71del+/+;Trp53f/f* 100-week-old case tissues. Invasive edges and delaminated islands have elevated NSD2 expression. Scale =100µm. F) Immunoblot of noted proteins in NSD2 wild-type (WT) and NSD2 knock-out (KO) class 1 (*R265-71del+/+;Trp53f/f;Cre+*) tumor organoids. G) Representative multiplex immunofluorescence images of AR and NSD2 in the NSD2 WT and KO tumor organoids. H) Representative images from NSD2 and AR PLA assay in NSD2 WT and KO class 1 tumor organoids. AR-NSD2 interaction is denoted by the pink dots. I) Area-based quantification of NSD2+/AR+ PLA foci from panel H (unpaired t-test). J) Growth curves (cell titer glow) of NSD2 WT and NSD2 KO Class 1 tumor organoids (n=6 biological replicates, two-sided t-test, mean with SD is shown). K) GSEA plots of AR up-regulated genes using the fold change from NSD2 KO (sgNSD2) vs WT tumor organoids. DEGS, differentially expressed genes. (n=2 biological replicates, GSEA enrichment test). L) Overlap of AR cistrome in NSD2 WT and KO class 1 mutant tumor organoids. M) Genomic location of AR at NSD2-dependent and independent sites defined from the overlap analysis in panel L. N) ChIP-seq read density heatmaps of AR at AR enhancers in NSD2 WT and KO class 1 tumor organoids. O) Top five known HOMER motifs present within NSD2-dependent and independent AR sites in class 1 mutant tumor organoids (HOMER, hypergeometric test). P) Box plots of normalized ChIP-seq reads of H3K36me2 at NSD2-dependent and NSD2-independent AR sites in class 1 mutant tumor organoids (n = top 1000 sites, two-sided t-test). Box plot center, median; box, quartiles 1-3; whiskers, quartiles 1-3 ± 1.5 × interquartile range; dot, outliers.

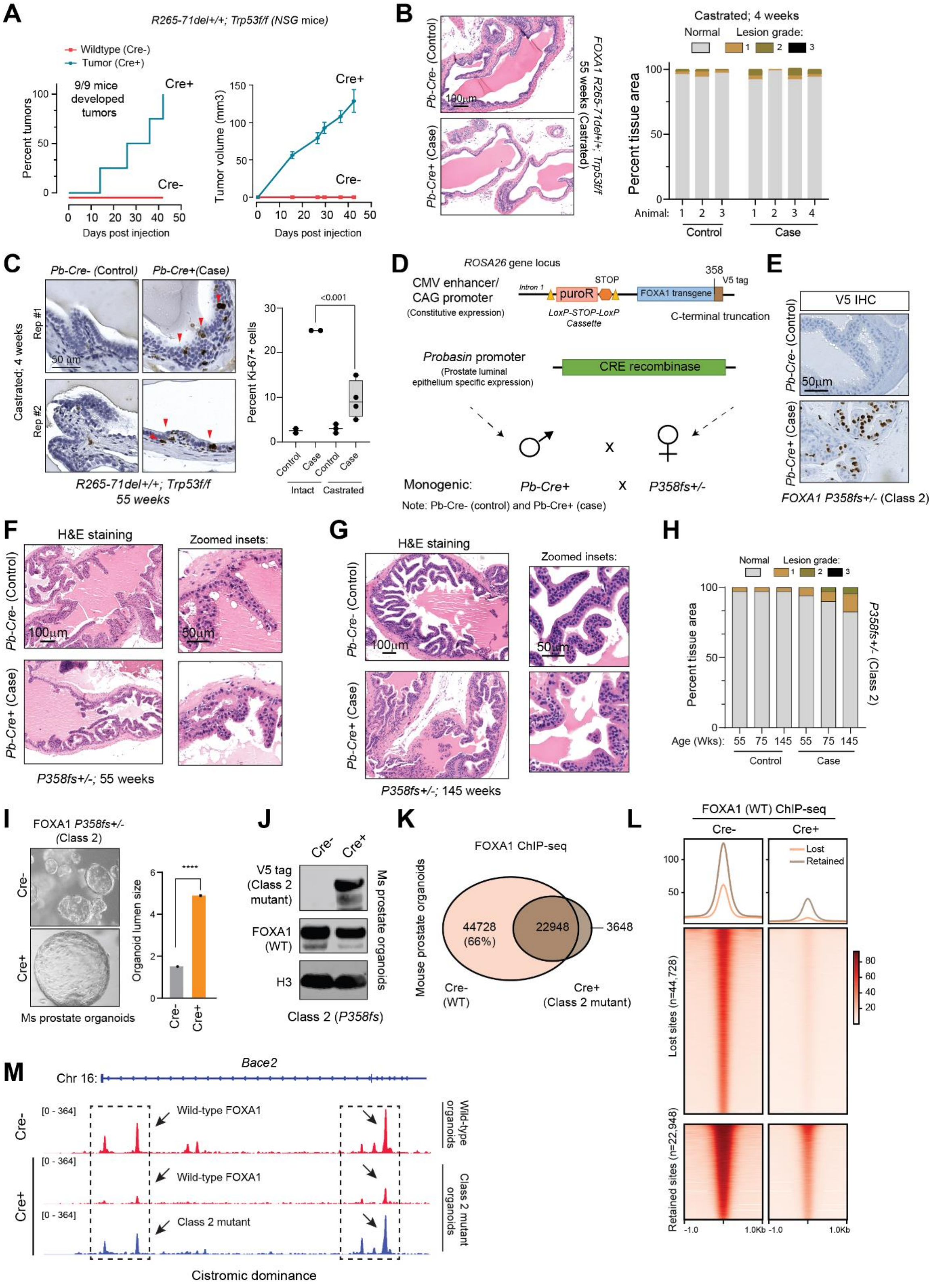

A
R265-71del+/+; Trp53f/f (NSG mice)
Wildtype (Cre-)
Tumor (Cre+)
9/9 mice developed tumors
Cre+
Cre-
Percent tumors
Days post injection
Tumor volume (mm3)
B
Pb-Cre- (Control)
Pb-Cre+ (Case)
FOXA1 R265-71del+/+; Trp53f/f
55 weeks (Castrated)
100µm
Castrated; 4 weeks
Normal
Lesion grade:
Percent tissue area
Animal:
Control
Case
C
Pb-Cre- (Control)
Pb-Cre+(Case)
Castrated; 4 weeks
Rep #1
Rep #2
50 µm
R265-71del+/+; Trp53f/f
55 weeks
<0.001
Percent Ki-67+ cells
Control
Case
Intact
Castrated
D
ROSA26 gene locus
CMV enhancer/
CAG promoter
(Constitutive expression)
Intron 1
STOP
puroR
LoxP-STOP-LoxP
Cassette
358
V5 tag
FOXA1 transgene
C-terminal truncation
Probasin promoter
(Prostate luminal
epithelium specific expression)
CRE recombinase
Monogenic:
Pb-Cre+
x
P358fs+/-
Note: Pb-Cre- (control) and Pb-Cre+ (case)
E
V5 IHC
Pb-Cre- (Control)
Pb-Cre+ (Case)
50µm
FOXA1 P358fs+/- (Class 2)
F
H&E staining
Zoomed insets:
Pb-Cre- (Control)
Pb-Cre+ (Case)
100µm
50µm
P358fs+/-; 55 weeks
G
H&E staining
Zoomed insets:
Pb-Cre- (Control)
Pb-Cre+ (Case)
100µm
50µm
P358fs+/-; 145 weeks
H
Normal
Lesion grade:
Percent tissue area
P358fs+/- (Class 2)
Age (Wks):
55
75
145
Control
Case
I
FOXA1 P358fs+/-
(Class 2)
Cre-
Cre+
Ms prostate organoids
Organoid lumen size
****
J
Cre-
Cre+
V5 tag
(Class 2
mutant)
FOXA1
(WT)
H3
Ms prostate organoids
Class 2 (P358fs)
K
FOXA1 ChIP-seq
Mouse prostate organoids
44728
(66%)
22948
3648
Cre-
(WT)
Cre+
(Class 2 mutant)
L
FOXA1 (WT) ChIP-seq
Cre-
Cre+
Lost
Retained
Lost sites (n=44,728)
Retained sites (n=22,948)
-1.0
1.0Kb
M
Bace2
Chr 16:
[0 - 364]
Wild-type FOXA1
Class 2 mutant
Wild-type organoids
Class 2 mutant organoids
Cistromic dominance

Fig. S7. FOXA1 Class 2 mutants cannot initiate pre-cancerous lesions. A) Left: Reverse Kaplan-Meier plot of subcutaneous organoid grafting of *R265-71del+/+;Trp53f/f* tumor organoids in *NSG* mice. Right: Tumor volumes of *R265-71del+/+;Trp53f/f* Cre- and Cre+ tumor organoids grafted in the subcutaneous flanks of *NSG* mice. B) Left: Representative H&E stained sections of control and case *R265-71del+/+;Trp53f/f* 55-week-old castrated prostate tissues. Scale =100µm.Right: Histopathological grading of tissues from the left panel. C) Left: Representative Ki67 IHC stained sections of control and case *R265-71del+/+;Trp53f/f* 55-week-old castrated prostate tissues. Red solid arrows highlight a few Ki67+ nuclei. Scale=50µm. Right: Percentage of Ki67+ cells in the *R265-71del+/+;Trp53f/f* 55-week-old intact and castrated case/control tissues (unpaired t-test). Box plot: center line, median; box, interquartile range (Q1–Q3); whiskers, minimum to maximum values; all individual data points shown. D) Schematic of *FOXA1* Class 2 (*P358fs*) knock-in transgenic mouse model and mating strategies for monogenic (*P358fs+/-*) mouse lines. Probasin-Cre (*Pb-Cre*) ensures prostate-specific expression of FOXA1. Control and case animals are denoted as *Pb-Cre-* and *Pb-Cre+,* respectively. E) Representative V5-epitope tag IHC in control (*Pb-Cre-*) and case (*Pb-Cre+*) Class 2 animals. Scale =50µm. F) Representative H&E-stained sections of 55-week-old FOXA1 *P358fs+/-* case and control tissues. Scale =100µm. G) Representative H&E-stained sections of 145-week-old FOXA1 *P358fs+/-* case and control tissues. Scale =100µm. H) Histopathological grading of 55, 75, and 145-week-old FOXA1 *P358fs+/-* case and control tissues. I) Left: Representative images of *FOXA1 P358fs+/-* Cre- and Cre+ mouse prostate organoids. Cre- (control) organoids lack expression of the Class 2 mutation, while Cre+ (case) organoids express the Class 2 mutation. Right: Lumen size of the Cre- and Cre+ FOXA1 *P358fs+/-* organoids (unpaired t-test). J) Immunoblots of noted proteins in the Cre- and Cre+ *FOXA1 P358fs+/-* organoids. C-terminal truncated FOXA1 Class 2 protein can be detected with the V5-epitope tag antibody. K) Overlap of the wild-type FOXA1 cistrome in the Cre- and Cre+ Class 2 mutant mouse prostate organoids. L) ChIP-seq read-density heatmaps of FOXA1 wild-type at lost and retained sites in Class 2 *P358fs+/-* Cre- and Cre+ organoids. M) ChIP-seq read-density tracks of the wild-type and Class 2 mutant in Cre- and Cre+ FOXA1 *P358fs+/-* mouse prostate organoids. The black dotted box highlights regions of cistromic dominance.

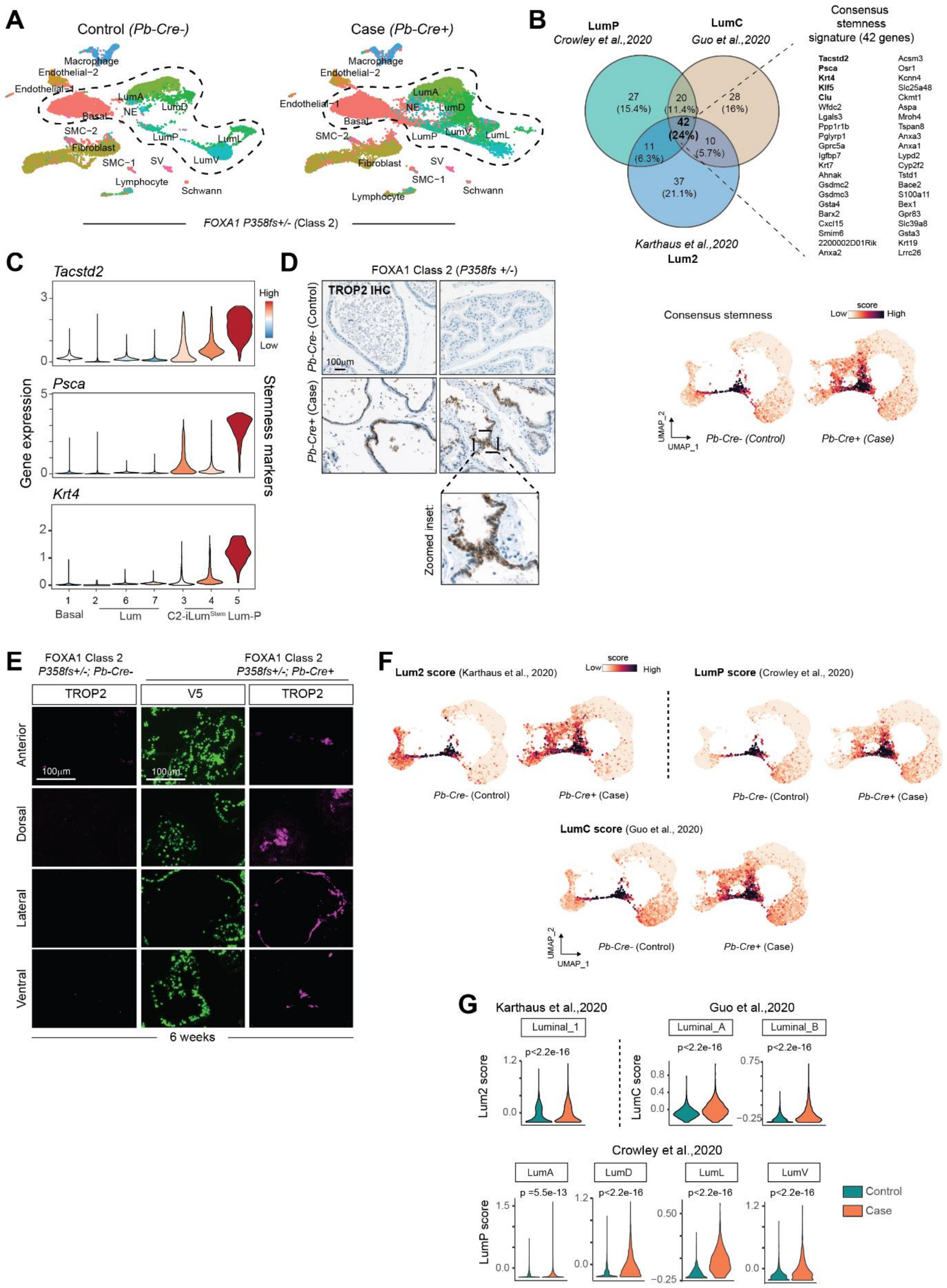
A
Control (Pb-Cre-)
Case (Pb-Cre+)
Macrophage
Endothelial−2
Endothelial−1
Basal
LumA
NE
LumD
LumP
LumL
LumV
SMC−2
Fibroblast
SMC−1
SV
Lymphocyte
Schwann
FOXA1 P358fs+/- (Class 2)
B
LumP
Crowley et al.,2020
LumC
Guo et al.,2020
Karthaus et al.,2020
Lum2
27 (15.4%)
20 (11.4%)
28 (16%)
42 (24%)
11 (6.3%)
10 (5.7%)
37 (21.1%)
Consensus stemness signature (42 genes)
Tacstd2
Psca
Krt4
Klf5
Clu
Wfdc2
Lgals3
Ppp1r1b
Pglyrp1
Gprc5a
Igfbp7
Krt7
Ahnak
Gsdmc2
Gsdmc3
Gsta4
Barx2
Cxcl15
Smim6
2200002D01Rik
Anxa2
Acsm3
Osr1
Kcnn4
Slc25a48
Ckmt1
Aspa
Mroh4
Tspan8
Anxa3
Anxa1
Lypd2
Cyp2f2
Tstd1
Bace2
S100a11
Bex1
Gpr83
Slc39a8
Gsta3
Krt19
Lrrc26
Consensus stemness
score
Low
High
UMAP_2
UMAP_1
Pb-Cre- (Control)
Pb-Cre+ (Case)
C
Tacstd2
Psca
Krt4
Gene expression
Stemness markers
High
Low
1
2
6
7
3
4
5
Basal
Lum
C2-iLumStem
Lum-P
D
FOXA1 Class 2 (P358fs +/-)
TROP2 IHC
Pb-Cre- (Control)
Pb-Cre+ (Case)
100µm
Zoomed inset:
E
FOXA1 Class 2
P358fs+/-; Pb-Cre-
FOXA1 Class 2
P358fs+/-; Pb-Cre+
TROP2
V5
TROP2
Anterior
Dorsal
Lateral
Ventral
100µm
6 weeks
F
Lum2 score (Karthaus et al., 2020)
LumP score (Crowley et al., 2020)
LumC score (Guo et al., 2020)
score
Low
High
Pb-Cre- (Control)
Pb-Cre+ (Case)
UMAP_2
UMAP_1
G
Karthaus et al.,2020
Guo et al.,2020
Luminal_1
Luminal_A
Luminal_B
p<2.2e-16
Lum2 score
LumC score
Crowley et al.,2020
LumA
LumD
LumL
LumV
p =5.5e-13
p<2.2e-16
LumP score
Control
Case

Fig. S8. Cistromically dominant FOXA1 Class 2 mutants activate stemness gene programs. A) Split UMAP plots of the distinct cell types from 10x single-cell RNA-sequencing data of FOXA1 *P358fs+/-* case and control animals. B) Top: Venn diagram showing the overlap of genes across three studies representing Luminal-P (*Crowley et al., 2020)*, Luminal-2 (*Karthaus et al., 2020*), and Luminal-C (*Guo et al., 2020*) stem-like clusters. The consensus stemness signature score is a 42-gene list. Bottom: Split UMAP plots with case or control tissue-derived cells colored by the consensus stemness score defined in the top panel. C) Violin plots of select stemness genes (single cell multiome gene expression, imputed) in the distinct Class 2 mutant case and control clusters. D) Representative TROP2 IHC in lateral and ventral prostatic lobes of FOXA1 *P358fs+/-* case and control tissue sections. Scale =100µm. E) Representative multiplex-IF of TROP2 and V5 in 6-week-old FOXA1 *P358fs+/-* case and control prostate lobes. Scale =100µm. F) Split UMAP plots of the case or control tissue-derived cells colored by the Lum2, LumP, and LumC scores. G) Violin plots of the LumP, LumC, and Lum2 scores in Class 2 mutant case and control luminal cells split by subpopulations annotated using three public datasets as reference (p-values obtained from Wilcoxon test).

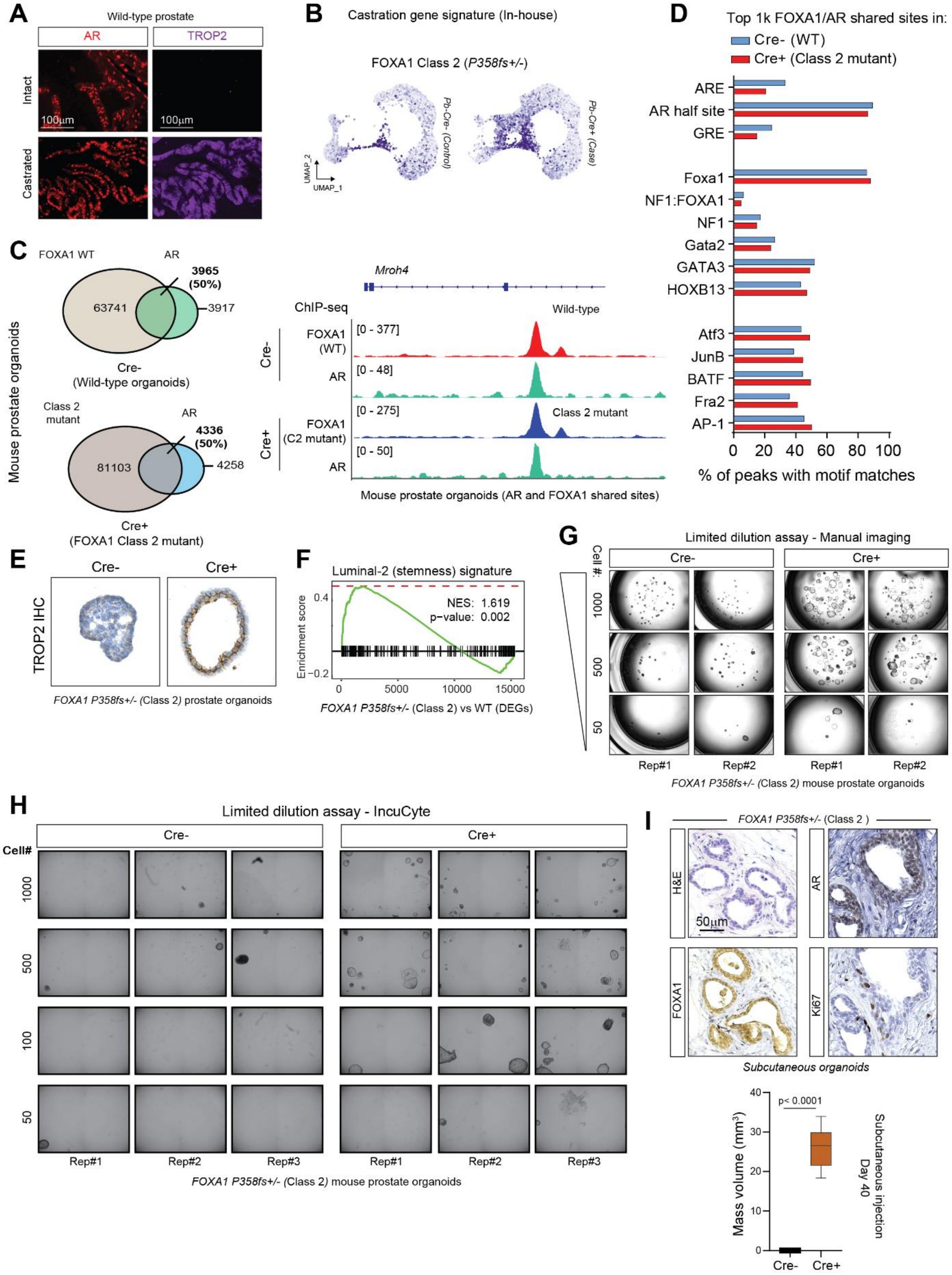
A
Wild-type prostate
AR
TROP2
Intact
Castrated
100μm
100μm
B
Castration gene signature (In-house)
FOXA1 Class 2 (P358fs+/-)
Pb-Cre- (Control)
Pb-Cre+ (Case)
UMAP_2
UMAP_1
C
Mouse prostate organoids
FOXA1 WT
AR
3965 (50%)
63741
3917
Cre-
(Wild-type organoids)
Class 2 mutant
AR
4336 (50%)
81103
4258
Cre+
(FOXA1 Class 2 mutant)
Mroh4
ChIP-seq
Wild-type
Cre-
FOXA1 (WT)
[0 - 377]
AR
[0 - 48]
Cre+
FOXA1 (C2 mutant)
[0 - 275]
Class 2 mutant
AR
[0 - 50]
Mouse prostate organoids (AR and FOXA1 shared sites)
D
Top 1k FOXA1/AR shared sites in:
Cre- (WT)
Cre+ (Class 2 mutant)
ARE
AR half site
GRE
Foxa1
NF1:FOXA1
NF1
Gata2
GATA3
HOXB13
Atf3
JunB
BATF
Fra2
AP-1
0 20 40 60 80 100
% of peaks with motif matches
E
Cre-
Cre+
TROP2 IHC
FOXA1 P358fs+/- (Class 2) prostate organoids
F
Luminal-2 (stemness) signature
NES: 1.619
p-value: 0.002
Enrichment score
0.4
−0.2
0 5000 10000 15000
FOXA1 P358fs+/- (Class 2) vs WT (DEGs)
G
Limited dilution assay - Manual imaging
Cell #:
Cre-
Cre+
1000
500
50
Rep#1 Rep#2 Rep#1 Rep#2
FOXA1 P358fs+/- (Class 2) mouse prostate organoids
H
Limited dilution assay - IncuCyte
Cre-
Cre+
Cell#
1000
500
100
50
Rep#1 Rep#2 Rep#3 Rep#1 Rep#2 Rep#3
FOXA1 P358fs+/- (Class 2) mouse prostate organoids
I
FOXA1 P358fs+/- (Class 2)
H&E
50μm
AR
FOXA1
Ki67
Subcutaneous organoids
p< 0.0001
Mass volume (mm3)
40
30
20
10
0
Cre- Cre+
Subcutaneous injection
Day 40

Fig. S9. FOXA1 Class 2 mutants drive higher stemness potential. A) Multiplex IF images of AR and TROP2 in intact and castrated wild-type mouse prostate tissues. Scale =100µm. B) Split UMAP plots of the case or control tissue-derived cells colored by the castration signature (defined in-house). C) Left: Overlap analysis of the AR cistrome with wild-type FOXA1 (top) and FOXA1 class 2 mutant (bottom) in Class 2 Cre- and Cre+ mouse prostate organoids. Right: ChIP-seq read-density tracks of AR and FOXA1 wild-type and Class 2 mutant proteins in FOXA1 *P358fs+/-* Cre- and Cre+ Class 2 organoids. D) Percentage of shared AR/FOXA1 wild-type sites or shared AR/FOXA1 class-2 mutant binding sites defined from panel c, containing known motifs of the labelled FOXA1 or AR cofactors. E) Representative TROP2 IHC positivity noted in FOXA1 *P358fs+/-* Cre- and Cre+ mouse prostate organoids. F) GSEA plots of the Luminal 2 up-regulated genes using the fold change from FOXA1 *P358fs+/-* Cre+ vs Cre- organoids. DEGS, differentially expressed genes. (n=2 biological replicates, GSEA enrichment test). G) Representative manual brightfield images of FOXA1 *P358fs+/-* Cre+ and Cre- organoids cultured in a limited dilution assay. Cells seeded per well are denoted on the left. H) Representative IncuCyte brightfield images of FOXA1 *P358fs+/-* Cre+ and Cre- organoids cultured in a limited dilution assay. Cells seeded per well are denoted on the left. I) Top: Representative H&E and AR, FOXA1, and Ki67 IHC stained sections in subcutaneous FOXA1 *P358fs+/-* Cre+ organoid masses formed in *CB17/SCID* mice. Scale =50µm. Bottom: Mass volumes of Cre- and Cre+ organoids as measured at Day 40 (unpaired t-test).

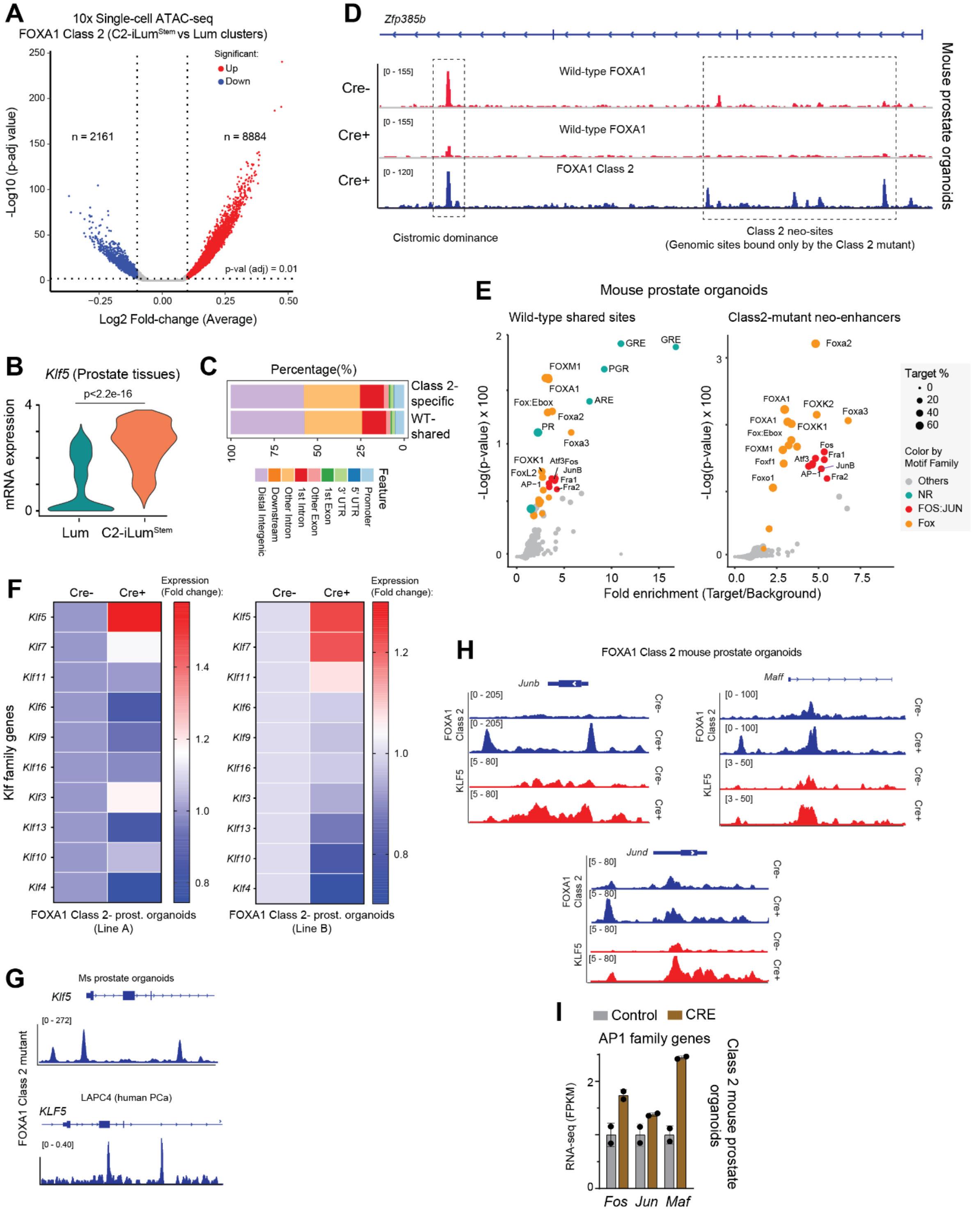
A
10x Single-cell ATAC-seq
FOXA1 Class 2 (C2-iLumStem vs Lum clusters)
Significant:
Up
Down
n = 2161
n = 8884
p-val (adj) = 0.01
-Log10 (p-adj value)
Log2 Fold-change (Average)
D
Zfp385b
Cre-
Cre+
Cre+
Wild-type FOXA1
Wild-type FOXA1
FOXA1 Class 2
Mouse prostate organoids
Cistromic dominance
Class 2 neo-sites
(Genomic sites bound only by the Class 2 mutant)
B
Klf5 (Prostate tissues)
p<2.2e-16
mRNA expression
Lum
C2-iLumStem
C
Percentage(%)
Class 2-specific
WT-shared
Feature
Promoter
5' UTR
3' UTR
1st Exon
Other Exon
1st Intron
Other Intron
Downstream
Distal Intergenic
E
Mouse prostate organoids
Wild-type shared sites
Class2-mutant neo-enhancers
-Log(p-value) x 100
Fold enrichment (Target/Background)
Target %
Color by Motif Family
Others
NR
FOS:JUN
Fox
F
Cre-
Cre+
Expression (Fold change):
Klf family genes
Klf5
Klf7
Klf11
Klf6
Klf9
Klf16
Klf3
Klf13
Klf10
Klf4
FOXA1 Class 2- prost. organoids (Line A)
FOXA1 Class 2- prost. organoids (Line B)
G
Ms prostate organoids
Klf5
FOXA1 Class 2 mutant
LAPC4 (human PCa)
KLF5
H
FOXA1 Class 2 mouse prostate organoids
Junb
Maff
Jund
FOXA1 Class 2
KLF5
I
Control
CRE
AP1 family genes
RNA-seq (FPKM)
Fos
Jun
Maf
Class 2 mouse prostate organoids

Fig. S10. FOXA1 Class 2 mutants pioneer neo-enhancers co-bound by KLF5 and AP-1 family proteins. A) Volcano plot showing differentially accessible (upregulated and downregulated) ATAC-peaks from 10x single-cell multiome ATAC seq of FOXA1 *P358fs+/-* control and case-specific clusters (logistic regression test). B) Violin plot of *Klf5* expression in Class 2 mutant tissues (single-cell multiome gene expression, Wilcoxon test). C) Genomic location of wild-type-shared and Class 2 mutant binding sites defined from the FOXA1 ChIP-seq in mouse prostate organoids. D) ChIP-seq read-density tracks of the wild-type and Class 2 mutant in Cre- and Cre+ FOXA1 *P358fs+/-* mouse prostate organoids. The black dotted box highlights the Class 2 neo-sites, which are genomic regions bound explicitly by FOXA1 Class 2 mutant. E) Fold change and significance of HOMER motifs enriched within the wild-type shared sites and Class 2 neo-sites from FOXA1 ChIP-seq in mouse prostate organoids (HOMER, hypergeometric test). F) Fold-change heatmap of the *Klf* family of genes in two independent FOXA1 *P358fs+/-* Cre- and Cre+ mouse prostate organoid lines. G) ChIP-seq read-density tracks of the FOXA1 Class 2 mutant within the *Klf5* locus in Cre+ Class 2 mouse prostate organoids (top) and the human LAPC4 PCa cell line (bottom). H) ChIP-seq read-density tracks of the FOXA1 Class 2 mutant and KLF5 within noted genes in FOXA1 *P358fs+/-* Cre- and Cre+ Class 2 organoids. I) Barplots showing RNA-seq expression of AP-1 family genes in Class 2 Cre- and Cre+ mouse prostate organoids (RNA-sequencing, n=2 replicates).

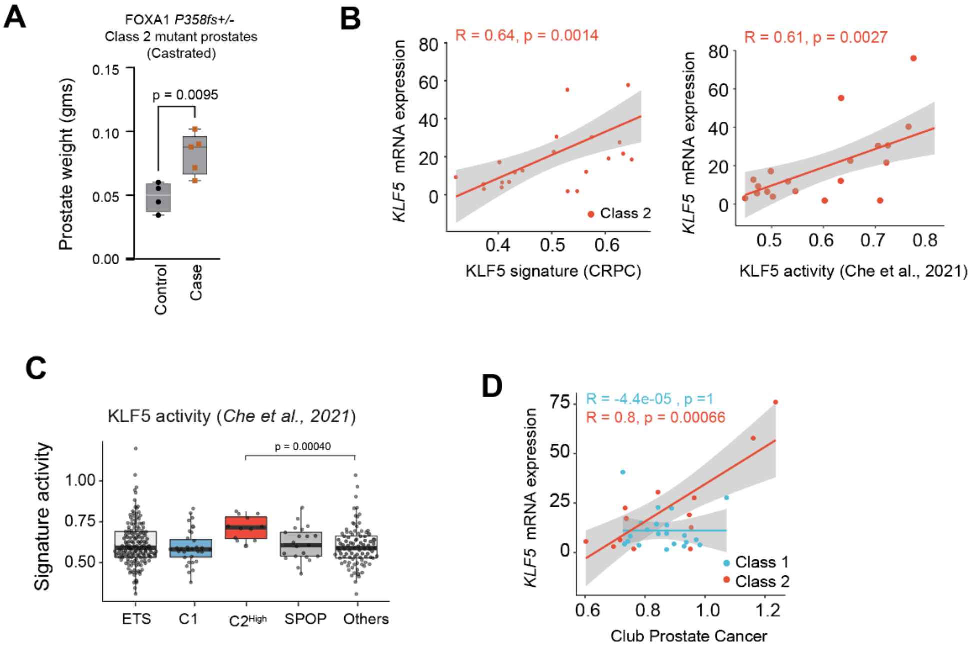

Fig. S11. FOXA1 Class 2 mutants activate KLF5 in clinical datasets. A) Boxplots of prostate weights from FOXA1 *P358fs+/-* control (n=4) and case (n=5) castrated prostate tissues (unpaired t-test). B) Correlation plots of *KLF5* transcript expression and two distinct KLF5 gene signatures (defined in Che et al.,2021) in FOXA1 Class 2 CRPC tissues (SU2C, n=371, pearson correlation test). C) Boxplots showing the KLF5 gene signature (defined in Che et al., 2021) in CRPC patient tissues (SU2C, n=371 patients) across distinct genomic driver groups (Wilcoxon rank-sum test). D) Correlation plots of *KLF5* transcript expression and a "Club Prostate Cancer score" in FOXA1 Class 2 CRPC tissues from the SU2C cohort (n=371 patients, pearson correlation test).

Table S1. (separate file)
FOXA1 Class-specific alterations in the Chinese Prostate Cancer Genome and Epigenome Atlas (CPGEA).

Table S2. (separate file)
Putative FOXA1 Class 1 mutant gene targets in mouse prostate organoids.

Table S3. (separate file)
Cell filtering cutoffs used in this study for single-cell RNA-sequencing and single-cell multiome (RNA+ATAC) sequencing.
